# Identification of distinct and shared biomarker panels in different manifestations of cerebral small-vessel disease through proteomic profiling

**DOI:** 10.1038/s43587-026-01081-7

**Published:** 2026-02-24

**Authors:** Ines Hristovska, Alexa Pichet Binette, Atul Kumar, Malin Wennström, Chris Gaiteri, Linda Karlsson, Olof Strandberg, Shorena Janelidze, Danielle van Westen, Erik Stomrud, Sebastian Palmqvist, Rik Ossenkoppele, Niklas Mattsson-Carlgren, Jacob W. Vogel, Oskar Hansson

**Affiliations:** 1 Clinical Memory Research Unit, Department of Clinical Sciences Malmö, Lund University, Lund, Sweden; 2 Department of Physiology and Pharmacology, Université de Montréal, Montréal, Québec Canada; 3 Centre de Recherche de l’Institut Universitaire de Gériatrie de Montréal, Montréal, Québec Canada; 4 Bioinformatics Core Facility, Biomedical Unit, Department of Medicine, Faculty of Health Sciences, University of Eastern Finland, Kuopio, Finland; 5 Cognitive Disorder Research Unit, Department of Clinical Sciences Malmö, Lund University, Lund, Sweden; 6 Department of Psychiatry, SUNY Upstate Medical University, Syracuse, NY USA; 7 Rush University Alzheimer’s Disease Center, Rush University, Chicago, IL USA; 8 Diagnostic Radiology, Department of Clinical Sciences Lund, Lund University, Lund, Sweden; 9 Imaging and Function, Skåne University Hospital, Lund, Sweden; 10 Memory Clinic, Skåne University Hospital, Malmö, Sweden; 11 Alzheimer Center Amsterdam, Neurology, Vrije Universiteit Amsterdam, Amsterdam UMC location VUmc, Amsterdam, The Netherlands; 12 Amsterdam Neuroscience, Neurodegeneration, Amsterdam, The Netherlands; 13 Department of Neurology, Skåne University Hospital, Lund, Sweden; 14 Wallenberg Center for Molecular Medicine, Lund University, Lund, Sweden; 15 Department of Clinical Sciences, Malmö, SciLifeLab, Lund University, Lund, Sweden

**Keywords:** Biomarkers, Cerebrovascular disorders

## Abstract

The pathophysiology underlying various manifestations of cerebral small-vessel disease (cSVD) remains poorly understood. Using high-throughput proteomics, we identified common and distinct proteomic signatures of white matter lesions (WMLs), microbleeds, infarcts and their subtypes, measured in 1,670 living patients. Across all cSVD manifestations, markers of extracellular matrix dysregulation and vascular remodeling were increased, including ELN, POSTN, CCN2 and especially MMP12, implicating endothelial and smooth muscle cells of the brain. These proteins were validated in cerebrospinal fluid from two additional datasets, and a subset detected in plasma predicted future cerebrovascular events in the UK Biobank better than risk scores currently used in clinical practice. Analysis focusing on WMLs found microglial-associated proteins associated with faster WML progression, whereas specific neuron-derived proteins mediated the link between WMLs and longitudinal cognitive decline. These data provide a comprehensive atlas of cSVD biomarkers, and our findings provide a promising roadmap for future diagnostics and therapeutics.

## Abstract (web summary)

High-throughput proteomics reveals shared and distinct protein signatures of cerebral small-vessel disease, linking vascular, immune and neuronal pathways to its pathophysiology, and identifying plasma proteins predicting cerebrovascular events.

## Main

Manifestations of cSVD have emerged as the leading vascular contributors to cognitive decline and subsequent dementia^1^, and as major risk factors for stroke^2^. cSVD can act as both a primary etiology and as a secondary pathology in numerous neurodegenerative conditions. These manifestations stem from a variety of pathological processes that affect the brain’s small arteries, arterioles, venules and capillaries, leading to structural and functional abnormalities within the brain’s microvascular network^3^. Although lifestyle changes and controlling vascular risk factors can reduce the risk for cSVD^4^, the development of more targeted preventative and therapeutic strategies is impeded due to the relatively limited understanding of the molecular pathophysiology of cSVD.

At present, clinical detection of cSVD relies strongly on magnetic resonance imaging (MRI), which identifies markers such as WMLs, subcortical infarcts and microbleeds^5^. Neuropathological studies and model systems have helped to characterize isolated pathways contributing to the etiology of cSVD^6–8^, but the larger landscape and timeline of altered pathways in living humans has been elusive. While existing literature offers insights into pathophysiological mechanisms for cSVD, including endothelial dysfunction, blood–brain barrier (BBB) breakdown and inflammation, these findings have not been consistently replicated, requiring further validation across larger cohorts with a broader spectrum of markers^9,10^. Associative evidence exists linking certain lesions together and with other diseases, but ultimately, we have little insight into their underlying etiology^11^. In this context, it is also important to understand how these cSVD-specific changes compare to broader cerebrovascular disease (CVD), such as those associated with large vessel disease^12^. Additionally, MRI lesions related to cSVD pathology are likely indicative of later stages in the pathological processes, underscoring the necessity of investigating the early changes that may precede these visible abnormalities. Such gaps in our knowledge emphasize the need for investigation using richer biomarker panels and larger cohorts to provide deeper insight into the pathophysiological processes involved in cSVD. Discovering robust markers and defining molecules related to earliest stages of disease will be crucial to the development and monitoring of therapeutic interventions.

Recent progress in highly sensitive and high-throughput protein measurement techniques now allow for a comprehensive large-scale proteomic profiling in cerebrospinal fluid (CSF) and blood^13–15^. These advances enable identification of potential biomarkers and delineate molecular mechanisms associated with disease pathogenesis in vivo^16,17^. In our study, we utilized the proximity extension assay (Olink) proteomics technology to extensively assess pathophysiological pathways concurrent with cSVD manifestations in CSF of several large population and patient cohorts. Our principal aims were (1) to identify both common and unique CSF proteome signatures associated with increased elevated burden, microbleeds and infarcts, and compare profiles according to etiology; (2) to provide in-depth characterization of the biological underpinnings of those signatures; (3) to identify proteins implicated in WML progression and mediating WML-associated cognitive decline; (4) to validate novel biomarkers in CSF and plasma cohorts; and (5) to assess their utility in plasma in predicting future cerebrovascular events in a large independent population-based study.

## Results

**Extended Data Fig. 1:**
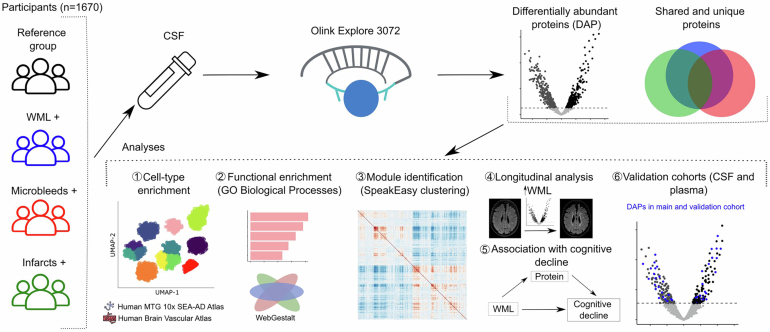
Overview of the study design. Legend: Cerebrospinal fluid (CSF) protein levels were quantified using Olink proteomics antibody-based Proximity Extension Assay (PEA) technology in 1670 participants from the BioFINDER-2 study. We analyzed differential protein abundance in CSF across various cSVD manifestations, namely white matter lesions (WMLs), microbleeds, and infarcts. Subsequently, for proteins identified as differentially abundant, cell-type specific enrichment was assessed using the Expression Weighted Cell Type Enrichment (EWCE) approach (1). Additionally, functional enrichment was performed utilizing Gene Ontology (GO) databases (2). Protein co-abundance modules were identified using a clustering algorithm, to explore the association between these biological modules and cSVD manifestations (3). Longitudinal analyses were performed to evaluate the association of Olink proteins with WML progression (4). Mediation analysis was conducted to identify whether WML-associated proteins mediate the relationship between WML and cognitive decline (5). Validation was performed in the same cohort using plasma, and in three distinct cohorts using CSF and plasma samples, and their utility in predicting future cerebrovascular events was assessed (6). Abbreviations: WML=White Matter Lesions, CSF=Cerebrospinal Fluid, GO=Gene Ontology.

We analyzed CSF protein levels for 2,943 proteins in 1,670 participants from the Swedish BioFINDER-2 cohort, ranging from cognitively unimpaired individuals to patients with mild cognitive impairment and dementia (Extended Data Fig. 1). Participants were stratified into groups reflecting the presence or absence of CVD, namely WML, microbleeds or infarcts. We distinguished 1,113 participants with absent/low WML burden and 557 with an increased WML burden, as defined by higher WML volumes (Supplementary Fig. 1). Similarly, individuals were classified based on the presence of microbleeds (1,350 without and 269 with ≥1 microbleed) and infarcts (1,445 without and 225 with ≥1 infarct). Supplementary Table 1 indicates associations between the presence of each CVD marker and various demographic and clinical measures. As expected, participants with CVD manifestations tended to be older, were more likely male, more likely demented and were more likely diagnosed with vascular risk factors compared to those without CVD.

### Existence of shared and distinct proteomic signatures in different CVD manifestations

**Fig. 1:**
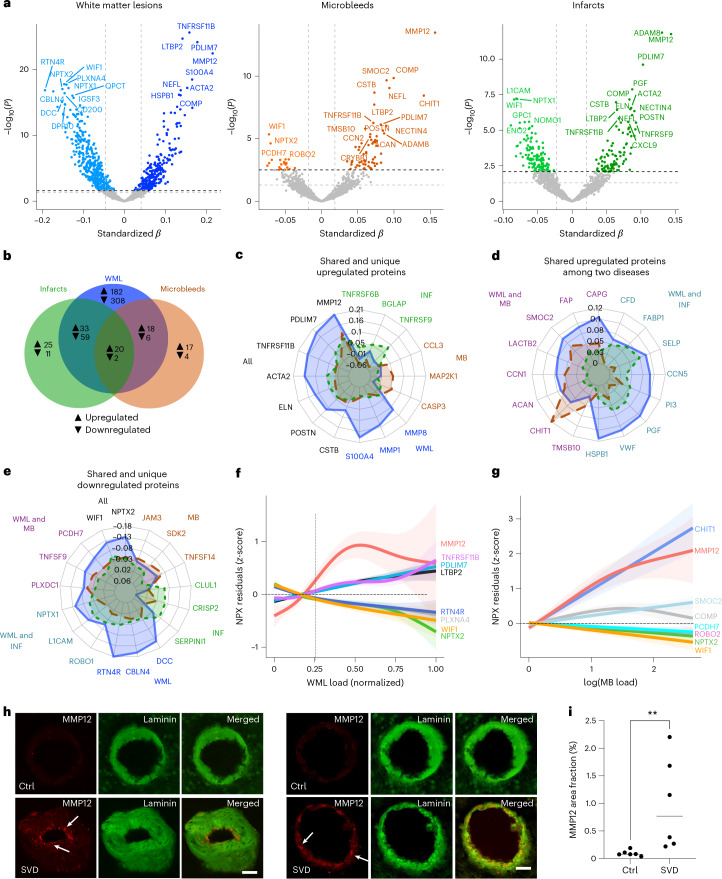
Differential protein expression in individuals with increased WML burden, microbleeds and infarcts. **a**, Volcano plots showing DAPs when comparing individuals with and without specific cSVD imaging markers: increased WML burden, microbleeds and infarcts. The models are adjusted for age, sex and average protein levels. The dashed lines represent significance threshold at *α* = 0.05 before (gray) and after (black) FDR correction. Proteins below the *P*_FDR_ < 0.05 threshold were considered significant. For clarity, only the top 20 proteins are labeled. All standardized *β* coefficients are derived from two-sided linear regressions. **b**, Venn diagram illustrating the shared and exclusive proteins associated with WMLs, microbleeds and infarcts, with proteins of increased abundance indicated in black and those with decreased abundance in gray. **c**–**e**, Radar charts depicting standardized β coefficients for top proteins, in relation to their association to disease: shared and disease-specific upregulated proteins (**c**); proteins commonly upregulated between two conditions (**d**); and shared and unique downregulated proteins (**e**). Each axis represents a different protein, with axis length indicating the standardized *β* coefficients magnitude for each condition. **f**,**g**, Proteins of interest from **a**, selected based on disease relevance or expression pattern, plotted against normalized WML load (**f**) and microbleed burden (**g**). Shaded areas correspond to the 95% CI. The vertical line indicates the cutoff for WML positivity. **h**, Representative images showing MMP12 (red, indicated by arrows) within laminin-positive (green) small arteries in control and SVD cases in the frontal lobe white matter tissue. **i**, Area fraction of MMP12 in arteries of six controls and six patients with SVD. Each point represents the mean MMP12 area fraction within laminin-stained regions from five arteries per case. Data were analyzed using a two-tailed Mann–Whitney *U*-test; **P = 0.0087. Scale bars, 50 µm. Source data

We first identified differentially abundant proteins (DAPs) related to broader CVD in individuals exhibiting increased WML burden (*n* = 707 proteins), microbleeds (*n* = 84) or infarcts (*n* = 228, false discovery rate-corrected *P* value (*P*_FDR_) < 0.05; Fig. 1a). As we aimed to identify and study proteins specifically associated with each of the three CVD manifestations, proteins were retained for further analysis only if their association with WMLs, microbleeds or infarcts, respectively, persisted when adjusting for the other two manifestations of CVD (for example, proteins associated with WMLs when adjusting for the presence of microbleeds and infarcts). Many proteins remained significant after adjusting for other CVDs (Fig. 1b). Relative to microbleeds and infarcts, a large part of the measured proteome exhibited alterations in the presence of WMLs. Summary statistics from the differential expression analyses can be found in Supplementary Table 2.

Several proteins, including MMP12, POSTN and ELN, showed a significant increase in abundance across all three manifestations examined of CVD independently (Fig. 1c). Notably, these proteins form a tightly connected cluster predominantly associated with extracellular matrix (ECM) organization (Supplementary Fig. 2), suggesting a shared vascular remodeling pathway involving ECM degradation, fibrosis and vessel wall instability; however, the expression of many proteins was found to be specific to particular imaging features of cSVD, most notably in WMLs. For instance, matrix metalloproteinases such as MMP1 and MMP8, were uniquely increased in WML. CCL3, involved in immune response, and CASP3, a key player in apoptosis, were specifically increased in microbleeds. On the other hand, several tumor necrosis factor receptor superfamily members, including TNFRSF9 and TNFRSF6B, were upregulated only in infarcts. This differential protein expression pattern with little shared upregulation between microbleeds and infarcts is in agreement with the hypothesis that these manifestations may have distinct underlying etiologies; however, some proteins were found to be increased in the context of WML as well as in either microbleeds or infarcts, as illustrated in Fig. 1d. A similar pattern was observed for proteins that were downregulated, with WIF1, involved in Wnt signaling and NPTX2, involved in synaptic function, consistently decreased across all forms of CVD. Compared to microbleeds and infarcts, many proteins were downregulated in the context of WML, with CBLN4 and RTN4R showing substantial negative associations (Fig. 1e).

We next sought to investigate how key proteins fluctuated in response to continuous (as opposed to binary) measures of WML (Fig. 1f) and microbleed (Fig. 1g) loads, respectively. Among the proteins that showed a gradual decrease, NPTX2 exhibited the most notable reduction as the severity of WML increased. MMP12 levels rose sharply with greater burden of both WMLs and microbleeds, with CHIT1 being more specific with increasing microbleed load.

Given the prominent role of MMP12 in cSVD, with strong associations observed across WML, infarcts and microbleeds, we further explored its expression using postmortem data (Fig. 1h). Our analysis revealed increased MMP12 expression within laminin-rich areas of small arteries in patients with cSVD compared to controls, strengthening the evidence of its involvement in the vascular pathology of cSVD (Fig. 1i). To complement these findings, we queried the DrugBank database^18^ and compiled druggability information for all significant proteins, including MMP12, offering insights into potential therapeutic targets (Supplementary Table 3).

### Stratification of CVD based on topography reveals distinct proteomic profiles

**Fig. 2:**
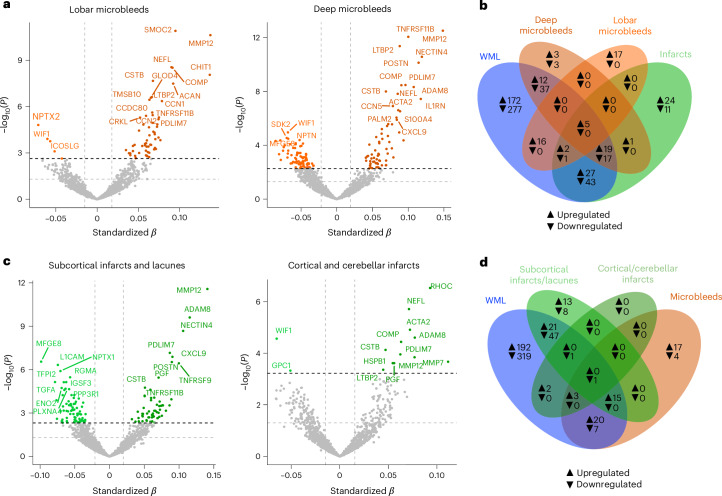
Differential protein expression stratified by CVD topography. **a**, Volcano plots showing DAP when comparing individuals with and without lobar and deep microbleeds. The models are adjusted for age, sex and average protein level. The dashed lines represent significance threshold at *α* = 0.05 before (gray) and after (black) FDR correction. Proteins below the *P*_FDR_ < 0.05 threshold were considered significant. For clarity, only the top 20 proteins are labeled. **b**, Venn diagram illustrating the shared and exclusive proteins associated with WMLs, deep microbleeds, lobar microbleeds and infarcts, with proteins of increased abundance indicated in black and those with decreased abundance in gray. **c**, Volcano plots showing DAPs when comparing individuals with and without subcortical infarcts/lacunes and cortical/cerebellar infarcts. The models are adjusted for age, sex and average protein level. For **a** and **c**, all standardized *β* coefficients are derived from two-sided linear regressions. **d**, Venn diagram illustrating the shared and exclusive proteins associated with WMLs, subcortical infarcts/lacunes, cortical/cerebellar infarcts and microbleeds, with proteins of increased abundance indicated in black and those with decreased abundance in gray. Source data

**Extended Data Fig. 2:**
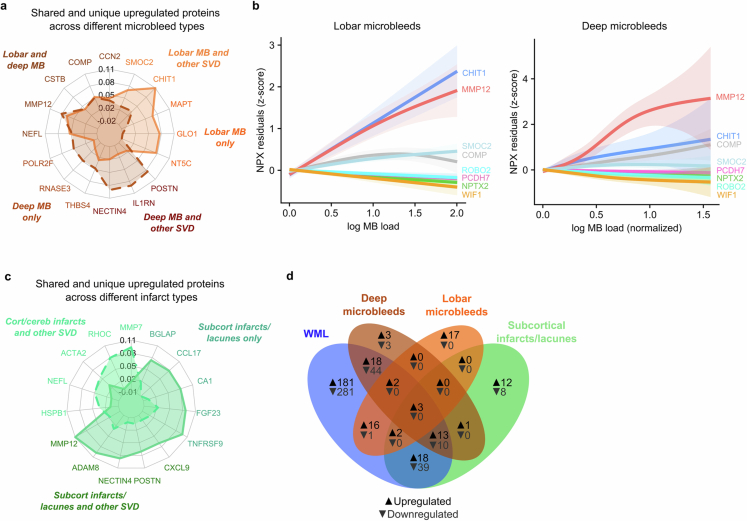
Characterization of proteomic profiles across different microbleed and infarct types. Legend: (**a**) Radar chart displaying standardized beta coefficients for top proteins across various microbleed types. Proteins shown may be either shared across different SVD subtypes or uniquely upregulated within microbleed subtypes. Each axis represents a different protein, with axis length indicating the magnitude of its standardized beta coefficient. (**b**) Top proteins associated with microbleeds (Fig. 1g), plotted against normalized lobar microbleed (**a**) and deep microbleed load (**b**). Solid line represents the fitted trend across pseudotime, and shaded areas correspond to the 95% confidence interval. (**c**) Radar chart displaying standardized beta coefficients for top proteins across various infarct types. Proteins shown may be either shared across different SVD subtypes or uniquely upregulated within infarct subtypes. Each axis represents a different protein, with axis length indicating the magnitude of its standardized beta coefficient. (**d**) Venn diagram illustrating the shared and exclusive proteins associated with WML, subcortical infarcts/lacunes, deep and lobar microbleeds, with proteins of increased abundance indicated in black and those with decreased abundance in gray. Source data

Building on our initial findings, we next stratified the data to investigate whether distinct proteomic signatures correspond to different topographies. Specifically, given that the topography of microbleeds is considered to reflect their underlying etiology, with deep microbleeds more associated with cSVD and lobar microbleeds with amyloid-β/cerebral amyloid angiopathy (CAA), we explored whether variations in their locations were mirrored by specific protein expression profiles (Fig. 2a,b). Our analysis identified distinct proteomic profiles associated with either deep or lobar microbleeds (Fig. 2a and Extended Data Fig. 2a). While most proteins were specific to one type, a few, such as MMP12, CSTB and COMP, were common to both deep and lobar microbleeds (Fig. 2b); however, these shared proteins were also significant in WML and infarcts, indicating they are not specific to microbleeds alone. Notably, CCL3, ITGB2 and GLOD4 were unique to lobar microbleeds, whereas THBS4, RNASE3 and BGLAP were exclusive to deep microbleeds. Of note, most DAP related to deep microbleeds overlapped with those associated with WMLs, and to a lesser extent, infarcts (a pattern not common for lobar microbleeds). Building on the increase in MMP12 and CHIT1 levels with escalating microbleed burden (Fig. 1g), we investigated whether this pattern differed between lobar and deep microbleeds. Our analysis revealed that MMP12 levels rose in both cases, whereas CHIT1 levels showed an increase comparable to MMP12 specifically with worsening lobar microbleed burden (Extended Data Fig. 2b).

Additionally, we separated infarcts into cortical/cerebellar infarcts, typically linked to large vessel disease and subcortical infarcts and lacunes, more associated with cSVD (Fig. 2c,d and Extended Data Fig. 2c). We identified a few significant proteins associated with cortical and cerebellar infarcts, with minimal overlap with subcortical infarcts and lacunes. Notably, RhoC, a key regulator of vascular homeostasis, emerged as a top hit linked to cortical and cerebellar infarcts. By contrast, subcortical infarcts and lacunes exhibited a proteomic profile more closely aligned with WML and, to a lesser extent, deep microbleeds, indicating a stronger association with cSVD (Extended Data Fig. 2d). Unique proteins associated with subcortical infarcts and lacunes included those involved in immune regulation and metabolism, such as members of the tumor necrosis factor superfamily, as well as C3, CA1 and CA3. This overlap highlights the closer relationship between deep microbleeds, subcortical infarcts/lacunes, and WML, in contrast to the distinct profile observed in cortical/cerebellar infarcts and lobar microbleeds. Additionally, very few proteins were downregulated in cortical/cerebellar infarcts and lobar microbleeds, further distinguishing them from other small-vessel disease (SVD)-associated manifestations.

**Extended Data Fig. 3:**
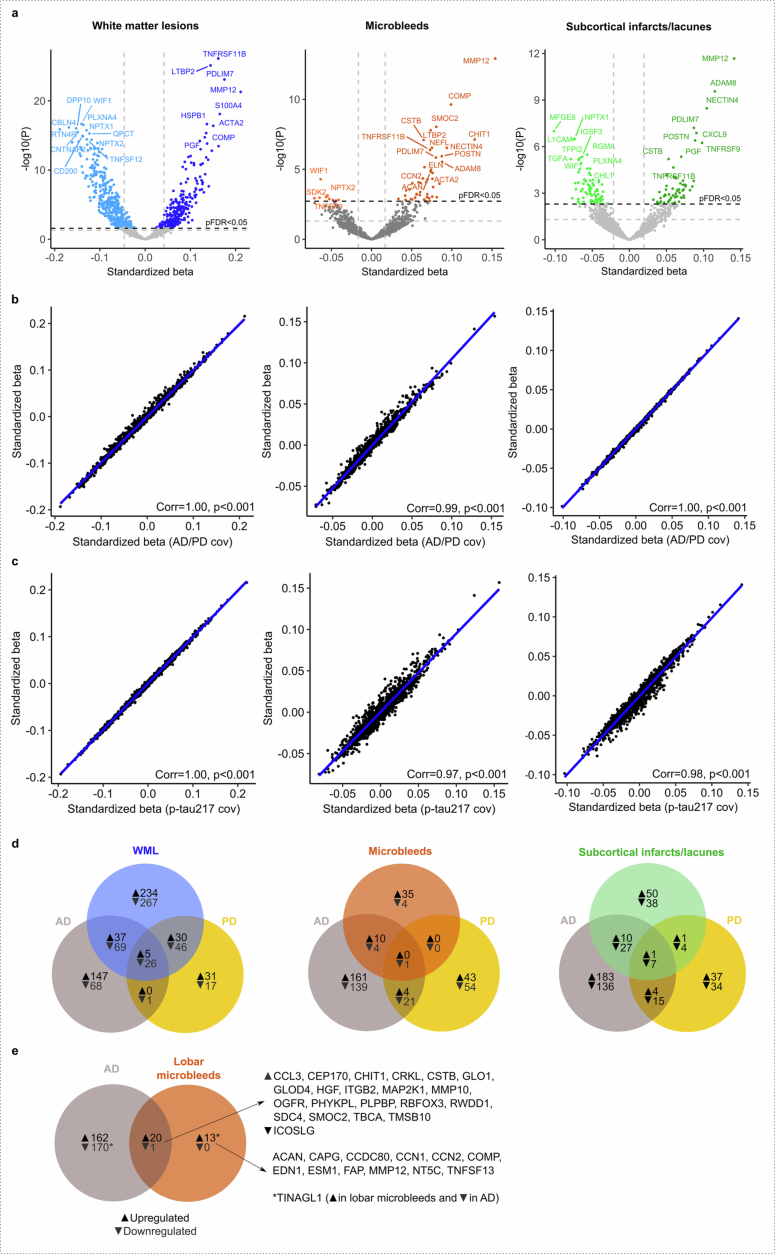
Differential protein expression in cSVD when models corrected for AD and PD diagnosis. Legend: (**a**) Volcano plot showing differentially abundant proteins (DAPs) when comparing individuals with and without a specific cSVD imaging feature while correcting for AD and PD diagnosis. The models were adjusted for age, sex, and average protein level. The dashed lines represent significance threshold at ɑ=0.05 before (gray) and after (black) FDR correction. Proteins below the p[FDR] < 0.05 threshold were considered significant. For clarity, only the top 20 proteins are labeled. All standardized β coefficients are derived from two-sided linear regressions. (**b**) Comparison of the standardized betas from the main analysis assessing differential protein abundance vs. the standardized betas when further adding AD and PD diagnosis as covariates. (**c**) Comparison of standardized betas from the main analysis of differential protein abundance versus the betas obtained when additionally adjusting for continuous CSF p-tau217. For (**b**) and (**c**), the shaded band denotes the 95% confidence interval around the fitted linear regression line. The exact p values can be found in Source data extended Fig. 3. (**d**) Venn diagram illustrating the overlap of differentially abundant proteins associated with all three cSVD imaging features and AD and PD, based on models mutually adjusted for the other conditions. (**e**) Venn diagram illustrating the overlap of differentially abundant proteins associated with lobar microbleeds and AD, based on models mutually adjusted for the other condition. Proteins are grouped by direction of association (upregulated or downregulated), with gene names shown alongside the diagram. Source data

To further explore the specificity of the identified proteomic signatures to cSVD, we assessed whether the associations observed for WMLs, microbleeds and subcortical infarcts and lacunes were independent of comorbid neurodegenerative disease. Effect sizes remained highly consistent after adjusting for comorbid Alzheimer’s disease (AD) or Parkinson’s disease (PD) as clinical diagnosis or using a continuous measure of AD pathology, with limited overlap with AD- or PD-associated proteins (Extended Data Fig. 3). Similar patterns were observed when infarcts were considered more broadly (Supplementary Fig. 3). As lobar microbleeds are considered a manifestation of CAA and are closely linked to AD, we examined which proteins remained significantly associated with lobar microbleeds after adjusting for the presence of AD and compared these to proteins associated with AD (Extended Data Fig. 3e). Several proteins, such as MMP12, CCN1, CCN2 and CCDC80, remained significantly associated with lobar microbleeds even after correction for AD and were also linked to other cSVD imaging markers. This supports their involvement in vascular-specific pathology rather than AD-related processes. Conversely, 21 proteins were significantly associated with both lobar microbleeds and AD, even after mutual adjustment, including ITGB2, TMSB10, SMOC2, GLOD4 and GLO1. These findings suggest the presence of shared mechanisms between lobar microbleeds and AD, particularly involving inflammation, matrix remodeling and cellular stress pathways. Although CAA cannot be directly measured in this cohort, the protein overlap and divergence between lobar microbleeds and AD suggest shared and distinct mechanisms, supporting CAA-related pathology while helping to disentangle vascular and neurodegenerative contributions.

To evaluate the robustness of our findings, we performed sensitivity analyses using stricter thresholds (for example, ≥2 lesions) and excluded those with only one lesion (Supplementary Fig. 4). We also analyzed infarcts and microbleeds as continuous (log-transformed) variables and tested alternative thresholds for defining WML burden. These approaches yielded highly correlated results with the original thresholds, supporting the consistency of our primary findings across lesion definitions. Furthermore, adjusting for vascular risk factors and dementia in a sensitivity analysis did not meaningfully change the results from the primary models (Supplementary Fig. 5). Of note, restricting this analysis to cognitively unimpaired individuals yielded strong correlations in effect sizes for WMLs and infarcts, but weaker correlations for microbleeds (Supplementary Fig. 6), suggesting that proteomic associations with microbleeds may be more sensitive to cognitive status.

To assess how our findings relate to the commonly used SVD score^19^, we applied a modified version incorporating WML, microbleeds, and lacunes (Supplementary Fig. 7). We found that many proteins associated with the individual markers were also associated with the composite score; however, a substantial number of WML-associated proteins were not, supporting the use of separate analyses to better capture lesion-specific proteomic signatures.

### cSVD is associated with greater neurovascular and lesser neuronal cellular responses

To better understand the specific cellular mechanisms underlying the different cSVD manifestations, we sought to uncover the cell-type specificity of DAPs and their biological roles. For cell-type specificity, we used expression weighted cell type enrichment (EWCE) on single-cell transcriptome data from the SEA-AD Atlas and the Human Brain Vascular Atlas, the latter providing greater granularity into vascular cells thought to underpin cSVD.

**Fig. 3:**
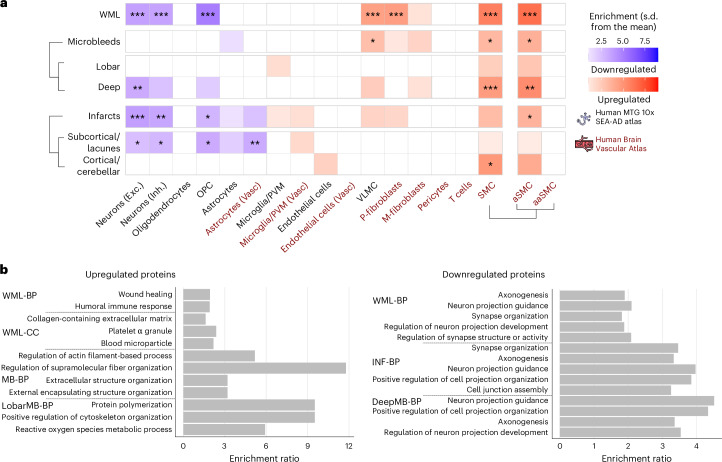
Cell-type and functional enrichment characterization of DAPs. **a**, Cell-type enrichment analyses for upregulated and downregulated proteins based on single-cell transcriptomics data from the Human MTG 10x SEA-AD and the Human Brain Vascular Atlas for different DAP categories using the EWCE package. The enrichment for upregulated proteins is shown in a palette of red, whereas the enrichment for downregulated proteins is shown in a palette of blue. The exact *P* values are in Source Data Fig. 3. **b**, Top five summary terms from functional enrichment analyses using GO databases (Biological Processes and Cellular Components) for different DAP categories. For cell-type and functional enrichment analyses, the 1,388 Olink proteins were used as background. *P_FDR_ < 0.05, **P_FDR_ < 0.01, ***P_FDR_ < 0.001. aSMC, arterial smooth muscle cell; aaSMC, arteriolar smooth muscle cell; BP, biological processes; CC, cellular components; IgC, integral component; IC, intrinsic component; INF, infarcts; M-fibroblast, meningeal fibroblast; MF, molecular functions; P-fibroblast, perivascular fibroblast; PM, plasma membrane; PVM, perivascular macrophage. Source data

Proteins upregulated across cSVD imaging markers, in WMLs and deep microbleeds, predominantly originated from vascular-associated cells, particularly vascular leptomeningeal cells (VLMCs) and smooth muscle cells (SMCs) (Fig. 3a). Further dissection of DAPs in SMC populations showed a significant enrichment specifically in arterial (as opposed to arteriolar) SMCs. To clarify the origin of this signal, we showed the expression levels of upregulated proteins in arterial SMCs, limited to those with >5% expression based on the Human Brain Vascular Atlas (Supplementary Fig. 8). Of note, WML-associated proteins were uniquely enriched in perivascular fibroblasts.

Conversely, downregulated proteins were predominantly associated with neuronal cells (both excitatory and inhibitory) as well as oligodendrocyte precursor cells (OPCs) for WML and subcortical infarcts/lacunes, indicating a distinct cellular response within the neuronal and oligodendrocyte populations. A similar trend was observed in deep microbleeds, though with significant enrichment only in excitatory neurons. Notably, only subcortical infarcts/lacunes exhibited significant enrichment of downregulated proteins in astrocytes, highlighting a unique cellular response to this cerebrovascular injury.

Building on these cellular insights, we sought to understand the biological significance of DAPs using functional enrichment analysis with the Gene Ontology (GO) database (Fig. 3b). All significant GO terms are listed in Supplementary Table 4. Upregulated proteins in WMLs highlight wound healing, immune responses and ECM remodeling, with significant overlap in the latter seen in both WMLs and microbleeds. Lobar microbleeds were uniquely associated with oxidative stress responses and protein polymerization. Conversely, downregulated proteins across WML, infarcts and deep microbleeds consistently pointed to disruptions in neuronal processes, such as axonogenesis and synapse organization, suggesting a common underlying neuronal and OPC impairment.

**Extended Data Fig. 4:**
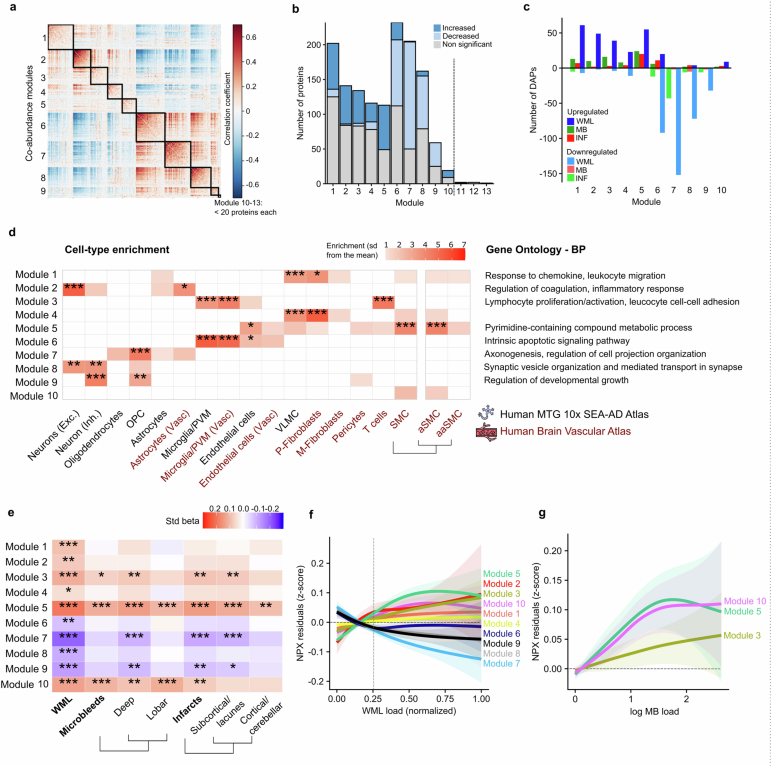
Clustering algorithm derived modules of protein co-abundance and their association with cSVD. Legend: (**a**) Heatmap depicting protein-protein correlation matrix within the Olink data. Black outlined boxes indicate co-abundance modules, with modules 10-13 comprising fewer than 20 proteins each. (**b**) Distribution of proteins across co-abundance modules. Bar heights represent the number of proteins in each module, categorized by changes in abundance in all three cSVD manifestations combined: increased (dark blue), decreased (pale blue), and non-significant (gray). Modules 11 to 13, demarcated by a dashed line, contain few proteins, and are not used in subsequent analysis. (**c**) Bar chart illustrating the module-wise distribution of DAP across various cSVD conditions. Upregulated proteins are represented above the horizontal axis, and downregulated proteins are shown below the axis in corresponding colors for each condition. (**d**) Heatmap displaying cell-type enrichment across protein co-abundance modules, using single-cell transcriptomics data from the Human MTG 10x SEA-AD and the Human Brain Vascular Atlas. Adjacent to the heatmap, key biological processes identified through Gene Ontology (GO) – Biological Processes (BP) enrichment analysis are listed for each module. The exact p values can be found in Source data extended Fig. 4. (**e**) Heatmap of standardized beta coefficients illustrating associations between biological modules and WML, microbleeds and infarcts. All standardized β coefficients are derived from two-sided linear regressions. (**f**, **g**) Trajectories of average protein expression within significant modules as a function of normalized (**f**) WML load and (**g**) microbleed load, showcasing the relationship between protein abundance and disease burden. Solid line represents the fitted trend across pseudotime, and shaded areas correspond to the 95% confidence interval. The vertical line indicates the cutoff for WML positivity. *corresponds to p_FDR_ < 0.05, **corresponds to p_FDR_ < 0.01, ***corresponds to p_FDR_ < 0.001. Source data

To further explore the molecular mechanisms underlying cSVD, we used the SpeakEasy2 (ref. ^20^) network-based clustering algorithm to identify co-abundance modules in CSF proteomics data, subsequently annotating these modules for cell-type specificity and biological processes (Extended Data Fig. 4a–d and Supplementary Table 5). The assignment of proteins to each module is found in Supplementary Table 2. Among the ten modules analyzed, those with increased protein abundance in SVD were primarily linked to vascular and immune responses (Extended Data Fig. 4e). Notably, module 5, enriched with proteins involved in metabolism, found in endothelial and arterial SMCs (for example, MMP12, PDLIM7 and ACTA2), showed a marked increase with WMLs and microbleeds even at lower burden levels, whereas module 3, associated with immune functions, steadily rose without plateauing at higher WML and microbleed loads (Extended Data Fig. 4f,g). Modules with decreased protein abundance were primarily associated with neuronal processes, enriched in proteins linked to OPC and inhibitory neurons, and involved in axonogenesis and developmental growth regulation. Additionally, WMLs were negatively correlated with module 8, which was enriched in proteins associated with synaptic vesicle organization and transport in both excitatory and inhibitory neurons. Thus, cSVD is associated with alterations in metabolic, vascular, immune and neuronal processes along the cSVD continuum.

### Contribution of immune-related proteins in the progression of WML over time

**Fig. 4:**
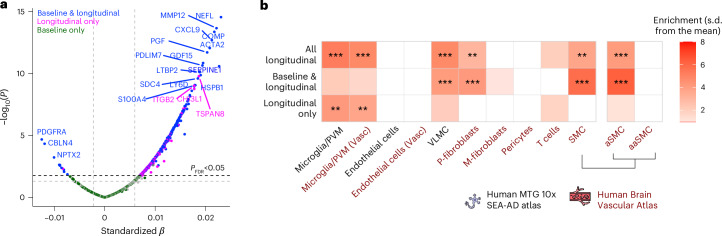
Differential abundance and cell-type enrichment in longitudinal WML progression. **a**, Volcano plots showing DAPs associated at baseline with WML progression over time. Linear mixed-effect models were adjusted for age, sex, average protein level and intracranial volume, with a protein × time interaction included in the model. The dashed lines represent *P* < 0.05 (gray) and *P*_FDR_ < 0.05 (black) significance thresholds, and proteins below the *P*_FDR_ < 0.05 threshold were considered significant. Protein color coding indicates significance relative to WMLs at baseline: proteins associated with WMLs both at baseline and longitudinally are highlighted in blue; those exclusively associated with longitudinal WMLs are in magenta, and proteins associated solely with WMLs at baseline are shown in dark green. For clarity, only the top upregulated and downregulated proteins from the first two groups are labeled. All standardized *β* coefficients are derived from two-sided linear mixed-effects models. **b**, Cell-type enrichment analyses based on single-cell transcriptomics data from the Human MTG 10x SEA-AD and the Human Brain Vascular Atlas for different DAP categories using the EWCE package. For cell-type enrichment analyses the 1,388 Olink proteins were used as background. Not all cell types are shown due to nonsignificant enrichment. Exact *P* values are shown in Source Data Fig. 4. *P_FDR_ < 0.05, **P_FDR_ < 0.01, ***P_FDR_ < 0.001. Source data

Our data expectedly showed an overall increase in WML volume over time in individuals with longitudinal follow-up (*n* = 856 individuals, average follow-up time of 2.94 ± 1.02 years; Supplementary Fig. 9). We proceeded in identifying the proteins at baseline that were associated with the rate of longitudinal increase in WML burden by applying linear mixed-effect models. This analysis revealed that abundance of several proteins at baseline was associated with faster WML progression over time (Fig. 4a and Supplementary Table 6). To gain a deeper insight into the role these proteins have in WML progression, we divided them into two categories: those associated with both baseline and longitudinal WML (shared, in blue), and those associated with longitudinal WML only (longitudinal-only, in magenta). Approximately half of the longitudinal WML-associated proteins were also associated with WML at baseline, indicating sustained upregulation, with NEFL, MMP12 and COMP being prominent among these proteins. By contrast, immune-related proteins, such as ITGB2 and CHI3L1 showed strong associations only with the rate of longitudinal WML progression, but not WML at baseline. In line with this observation, cell-type enrichment analysis indicated that shared proteins were significantly enriched in VLMCs (per the SEA-AD Atlas), as well as perivascular fibroblasts and SMCs (per the Human Vascular Atlas) (Fig. 4b). Proteins associated with WML progression that contribute to the arterial SMC signature are shown in Supplementary Fig. 10. Conversely, longitudinal-only proteins were significantly enriched in microglial and macrophage populations, suggesting important contribution of immune-related proteins in the progression of WMLs. Several nominally enriched biological processes aligned with key microglial functions, including immune response–regulating cell surface receptor signaling, extrinsic apoptotic signaling via death domain receptors, and cell redox homeostasis, pointing to roles in immune sensing, neuroinflammatory-induced cell death and oxidative stress regulation (Supplementary Table 7).

### Neuronal and OPC-associated proteins partly mediate the association between WML and cognitive decline

**Fig. 5:**
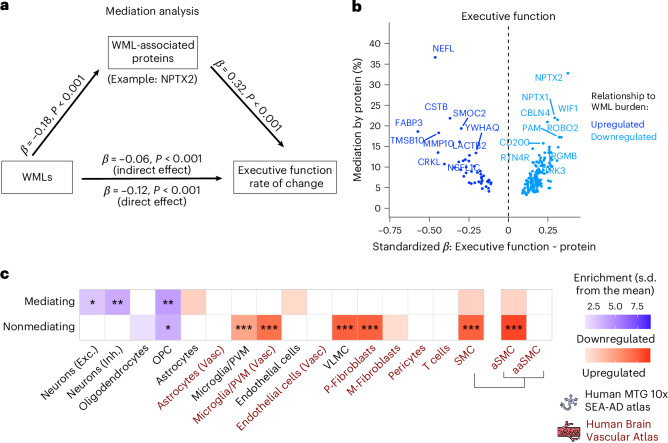
Characterization of WML-associated proteins mediating the relationship between WML and cognitive decline as measured by executive function. **a**, Path diagram of the mediation analysis model, using NPTX2 as a representative example of WML-associated proteins. **b**, Volcano plots showing the extent to which WML-associated proteins mediate the association between WMLs and executive function rate of change by standardized *β* values derived from the association between proteins and the executive function rate of change. The top ten upregulated and top ten downregulated proteins with the highest mediation effect are labeled. **c**, Comparison for cell-type enrichment between WML-associated proteins that mediate and those that do not mediate the interaction between WML and executive function rate of change based on single-cell transcriptomics data from the Human MTG 10x SEA-AD and the Human Brain Vascular Atlas using the EWCE package. For this analysis, the 1,388 Olink proteins were used as background. Exact *P* values are shown in Source Data Fig. 5. *P_FDR_ < 0.05, **P_FDR_ < 0.01, ***P_FDR_ < 0.001. Source data

**Extended Data Fig. 5:**
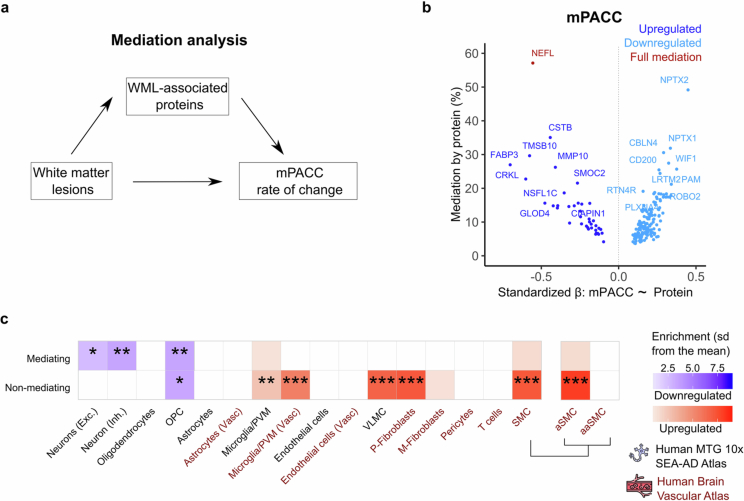
Characterization of WML-associated proteins mediating the relationship between WML and cognitive decline as measured by mPACC. Legend: (**a**) Path diagram of the mediation analysis models. (**b**) Volcano plots showing the extent to which WML-associated proteins mediate the association between WML and mPACC rate of change by the standardized beta values derived from the association between proteins and mPACC rate of change. The top 10 upregulated and top 10 downregulated proteins with highest mediation effect are labeled. (**c**) Comparison for cell-type enrichment between WML-associated proteins that mediate and those that do not mediate the interaction between WML and mPACC rate of change based on single-cell transcriptomics data from the MTG 10x SEA-AD Atlas and the Human Brain Vascular Atlas using the EWCE package. For this analysis, the 1,388 Olink proteins were used as background. The exact p values can be found in Source data extended Fig. 5. *corresponds to p_FDR_ < 0.05, **corresponds to p_FDR_ < 0.01, ***corresponds to p_FDR_ < 0.001. Abbreviations: OPC = oligodendrocyte precursor cells, PVM = perivascular macrophages, VLMC = vascular leptomeningeal cell, P-Fibroblasts=perivascular fibroblasts, M-Fibroblasts = meningeal fibroblasts, SMC = smooth muscle cell, aSMC = arterial smooth muscle cell, aaSMC=arteriolar smooth muscle cell. Source data

As WMLs are associated with cognitive decline, especially in executive functions (Fig. 5a), we next assessed the possible mediation of DAPs on the association between WMLs at baseline and longitudinal cognitive decline. We studied the impact of these proteins on changes in executive function (Trail Making Test-A (TMT-A), Trail Making Test-B (TMT-B) and the Symbol Digit Modalities Test (SDMT); Fig. 5 and Supplementary Table 8) and global cognition (modified Preclinical Alzheimer Cognitive Composite (mPACC); Extended Data Fig. 5). We found that proteins like CSTB and NEFL, which are upregulated in WMLs, alongside ECM and cell adhesion-related proteins (SMOC2, MMP10 and TMSB10), exhibited a significant partial mediation effect on rate of executive function decline, each contributing over 10% to the mediating effect (Fig. 5b). Similarly, key downregulated proteins associated with synaptic function, such as NPTX2, NPTX1 and CBLN4, were major contributors to this mediation. Cell enrichment analysis of proteins that were negatively associated with WML and that mediated cognitive decline showed that these were predominantly neuronal, including glutamatergic and GABA (γ-aminobutyric acid)-dependent (GABAergic) subtypes, and OPC-related (Fig. 5c). Proteins that had no mediating effect were primarily observed in OPCs. While proteins positively associated with WML and mediating cognitive decline did not exhibit significant cell-type enrichment, the nonmediating proteins were enriched in VLMCs, perivascular fibroblasts, arterial SMCs and microglial cells. These results were largely reproduced when looking at change in global cognition instead of executive function (Extended Data Fig. 5).

### CSF DAP are consistent across independent cohorts

**Fig. 6:**
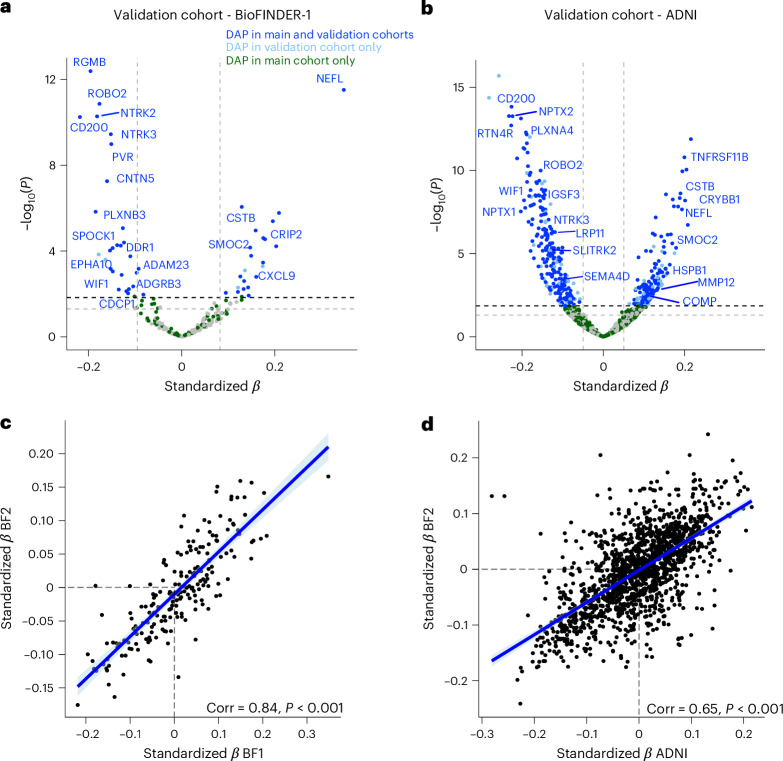
Validation of differentially abundant proteins in CSF in two independent cohorts. **a**,**b**, Volcano plots showing DAPs from the BioFINDER-1 validation cohort (**a**) or ADNI validation cohort (**b**), analyzed with WMLs as a continuous variable, adjusting for age, sex, intracranial volume and average protein level. Color coding reflects significance across cohorts: proteins significant in both the main and validation cohorts are shown in blue, those significant only in the validation cohort are depicted in pale blue and proteins significant solely in the main cohort are shown in green. Labels highlight the top 20 DAPs in BioFINDER-2. All standardized *β* coefficients are derived from two-sided linear regressions. **c**,**d**, Scatter-plots showing the correlations between standardized *β* values from linear regression analysis for proteins overlapping between BioFINDER-1 (**c**) or ADNI and BioFINDER-2 (**d**). The shaded band denotes the 95% CI around the fitted linear regression line. The exact *P* values can be found in Source Data Fig. 6. Source data

**Extended Data Fig. 6:**
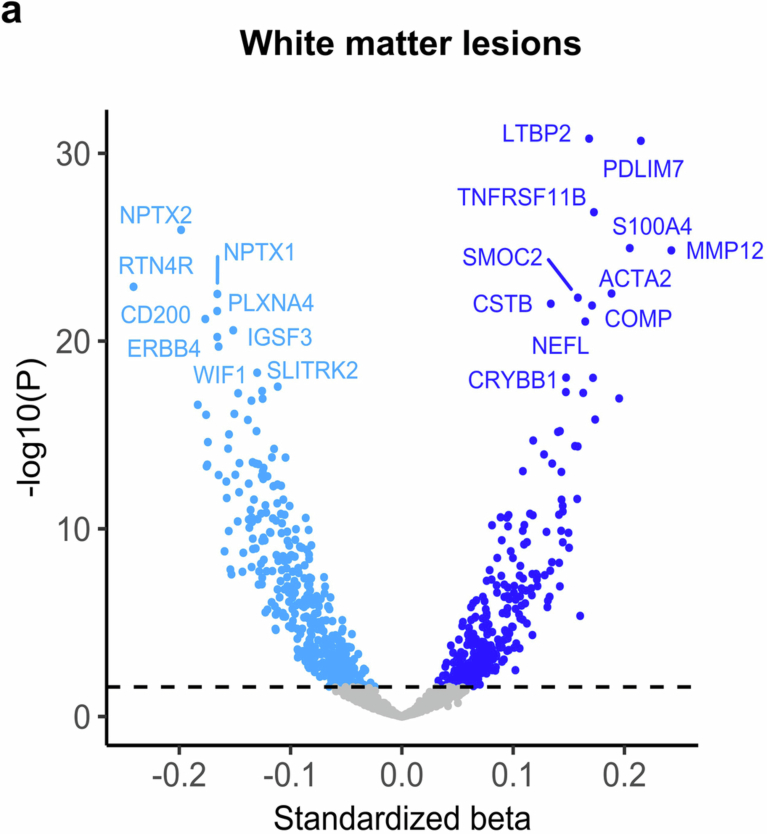
Differential protein expression related to continuous measures of WML volume. Legend: (**a**) Volcano plot showing differentially abundant proteins (DAPs), analyzed with WML as a continuous variable, and adjusting for age, sex, intracranial volume, and average protein level. The dashed lines represent significance threshold at ɑ=0.05 before (gray) and after (black) FDR correction. Proteins below the p[FDR] < 0.05 threshold were considered significant. For clarity, only the top 20 proteins are labeled. All standardized β coefficients are derived from two-sided linear regressions. Source data

To ensure robustness and generalizability of our findings regarding WML-associated DAPs, we sought validation in two additional independent cohorts with CSF proteomics data (using WML volume as a continuous variable; Supplementary Tables 9 and 10 and Extended Data Fig. 6). We first included 383 participants from the independent Swedish BioFINDER-1 study, all of whom had both CSF proteomics and MRI data. Our analysis focused on a subset of CSF proteins (*n* = 199) measured with Olink-based technology that overlapped with those examined in our BioFINDER-2 study. The second validation cohort was derived from the Alzheimer’s Disease Neuroimaging Initiative (ADNI), which included 729 participants, and used the aptamer-based SOMAscan 7K (v.4.1) platform. Linear regression models revealed a high degree of concordance in the differential protein abundance between the BioFINDER-1 and BioFINDER-2 cohorts, with a 68% agreement (Fig. 6a and Supplementary Table 11). Similarly, a 63% concordance was observed between the ADNI cohort and BioFINDER-2, with highly consistent direction and magnitude for the top proteins (Fig. 6b, Supplementary Table 12 and Supplementary Fig. 11). The correlation of standardized *β* values revealed robust associations, with a strong correlation coefficient of 0.84 between BioFINDER-1 and BioFINDER-2 (Fig. 6c). Additionally, a correlation of 0.65 between the ADNI cohort and BioFINDER-2 (Fig. 6d) indicated a high level of consistency in DAP patterns even in this cross-platform context.

### DAP in plasma predict future cerebrovascular events and infarcts in a population-based setting

**Fig. 7:**
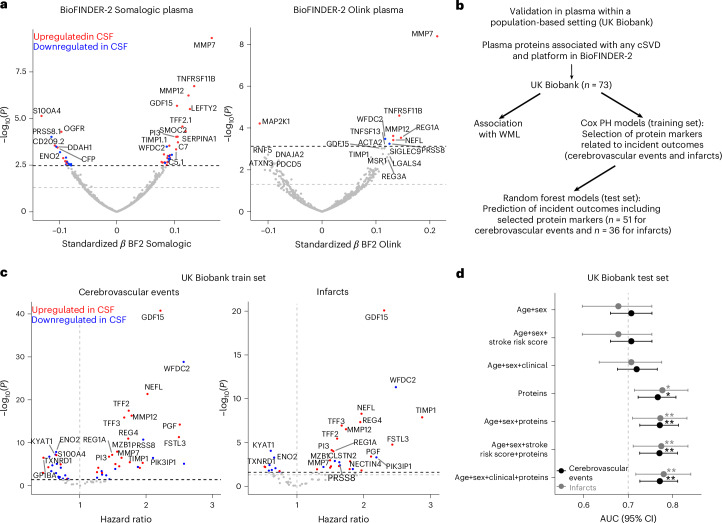
Validation of DAPs in plasma and their association with and predictive value for incident cerebrovascular outcomes. **a**, Volcano plots showing DAPs in plasma from the BioFINDER-2 cohort, assessed using Somalogic or Olink platform, analyzed with WMLs as a continuous variable, adjusting for age, sex, intracranial volume and average protein level. Labels highlight the top 20 DAPs. Significant proteins between the two analyses for all cSVD were retained for the UK Biobank analysis. **b**, Workflow for analysis in UK Biobank. **c**, Volcano plots illustrating prospective associations between the plasma cSVD set and the risk of cerebrovascular events or infarcts. The top 20 proteins are highlighted, with color coding indicating whether they were upregulated (red) or downregulated (blue) in CSF. For **a** and **c**, all standardized *β* coefficients were derived from two-sided linear regressions. **d**, Forest plot showing AUC for models predicting cerebrovascular events and infarcts, with points representing mean AUC values and horizontal bars indicating the 95% CI. The models were validated using a random forest classifier on the test dataset (*n* = 10,447 individuals). Different combinations of predictive features are presented, with a model incorporating age, sex and risk score (RS) as the base model. Outcomes (cerebrovascular events versus infarcts) are differentiated by point and line color. AUCs for the protein-based and combined models were compared to the baseline model using a two-sided DeLong’s test. The exact *P* values have been provided in Source Data Fig. 7. *ROC *P*_FDR_ < 0.05, **ROC *P*_FDR_ < 0.01. Source data

We next investigated whether CSF proteins associated with cSVD outcomes in BioFINDER-2 could be validated in plasma. Plasma proteomic analyses in BioFINDER-2 were conducted using the SOMAscan 7k platform (*n* = 1,599 individuals) and the Olink platform (*n* = 694 individuals). Several proteins were successfully validated in plasma for each cSVD outcome (Supplementary Tables 13 and 14). Notably, MMP7, MMP12, TNFRSF11B, GDF15, WFDC2 and NEFL were associated with WMLs in plasma (Fig. 7a). Of note, some proteins exhibited contrasting trends between CSF and plasma. For instance, S100A4, which had one of the highest standardized *β* coefficients for WMLs in CSF, was notably decreased in plasma. Conversely, WFDC2 showed the opposite pattern, being increased in plasma but reduced in CSF. Overall, we identified 90 proteins in plasma related to cSVD outcomes in BioFINDER-2, 73 of which were also present in the UK Biobank. This subset, referred to as the ‘plasma cSVD set’, was further investigated in the UK Biobank dataset in relation to its extensive data on cerebrovascular outcomes (Fig. 7b).

**Extended Data Fig. 7:**
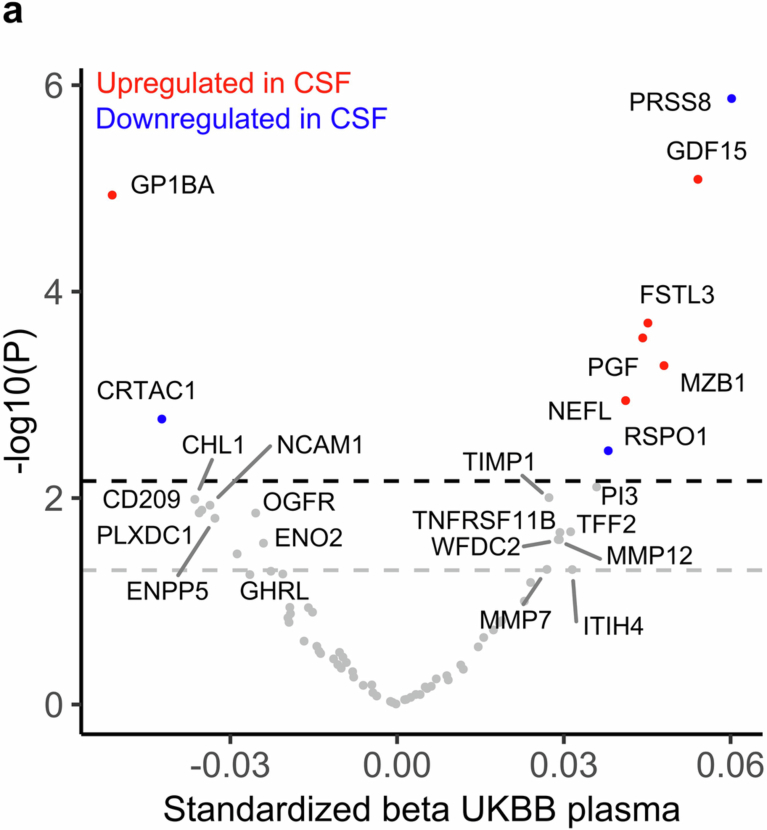
Validation of DAP in subjects with WML in the UK Biobank plasma dataset. Legend: (**a**) Volcano plots showing differentially abundant proteins (DAP) when comparing individuals with and without WML in the UK Biobank. The models are adjusted for age, sex, average protein level and time interval between MRI scans and CSF collection. The dashed lines represent significance threshold at ɑ=0.05 before (gray) and after (black) FDR correction. Proteins below the p[FDR] < 0.05 threshold were considered significant. For clarity, only the top 20 proteins are labeled. All standardized β coefficients are derived from two-sided linear regressions. Source data

We first examined the relationship of the 73-protein plasma set with WMLs, followed by an evaluation of whether these proteins were associated with the risk of cerebrovascular events or infarcts. Several key proteins from the plasma cSVD set, including GDF15, NEFL and PGF, were associated with WMLs in the UK Biobank (*P*_FDR_ < 0.05; Extended Data Fig. 7 and Supplementary Table 9). MMP12, MMP7 and TNFRSF11B showed nominal significance (*P* < 0.05) but did not pass FDR correction.

**Extended Data Fig. 8:**
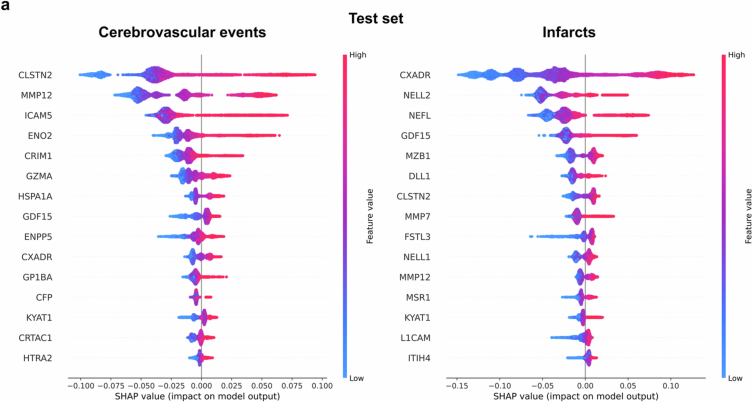
Shapley Additive exPlanations (SHAP) feature importance for cerebrovascular events and infarct prediction using Random forest classifier model. Legend: (**a**) SHapley Additive exPlanations (SHAP) feature importance for the model incorporating age, sex, clinical variables, proteins and average protein level for cerebrovascular events (left panel) and infarcts (right panel). For visualization purposes, we included only the top 15 features, shown in order of importance, from most important (top) to less important (bottom). Source data

Next, we divided the UK Biobank dataset into a training set (80%) for inferential analysis, and a left-out test set (20%) to use for prediction analysis on unseen data (Supplementary Table 15). In the training set, we used Cox proportional hazard ratios to identify the proteins in plasma associated with the risk of incident cerebrovascular outcomes over a 5-year period. Overall, 51 of the 73 proteins were significantly associated with cerebrovascular events, and 36 with infarcts (Fig. 7c and Supplementary Table 16). Among these, GDF15 emerged as a top hit in both analyses, with WFDC2, NEFL, PGF and MMP12 also identified as key proteins in both analyses. TIMP1 exhibited the highest hazard ratio for infarcts, whereas its association with any cerebrovascular events was comparatively weaker. We then evaluated if the combinations of those proteins (51 for cerebrovascular events and 36 for infarcts) could further improve the prediction of both outcomes in the test set using a random forest (Fig. 7d). We compared models with various feature combinations, including protein-only and protein-inclusive models, against a base model comprising age, sex and a clinical risk score (incorporating information from blood pressure, glucose and cholesterol levels, BMI, atrial fibrillation, diabetes, smoking and personal or family history of cerebrovascular events), the latter serving as benchmark in clinical practice (the American Heart Association Stroke Risk Assessment Score). Additionally, we evaluated models using the clinical variables that comprise the risk score, analyzed as continuous or binary variables. Across all feature sets, the addition of proteins significantly improved predictive performance beyond the base model (DeLong test, *P* < 0.05). For cerebrovascular events, the base model had an area under the curve (AUC) of 0.72 (95% CI 0.68–0.77), which improved to 0.77 (0.72–0.81) with the addition of proteins alone and remained around 0.78 (0.726–0.81) when age, sex and clinical covariates were added alongside proteins. For infarcts, the base model had an AUC of 0.68 (95% CI 0.60–0.75), increasing to 0.78 (0.71–0.83) with proteins alone, and reaching 0.78 (0.72–0.84) with the addition of age, sex and clinical covariates. Several proteins, such as GDF15 and MMP12, demonstrated high SHAP values, indicating their strong contribution to the model’s predictive performance for cerebrovascular events and infarcts (Extended Data Fig. 8). Notably, in the model combining age, sex, clinical variables and proteins, only protein features were included in the top 15 most important features for either model.

## Discussion

This study thoroughly investigates CSF proteomic alterations in various cSVD manifestations in several large cohorts, while also drawing parallels with broader CVD, including large artery disease. Earlier work focused primarily on isolated cSVD manifestations, often centered on WMLs, with limited protein panels assessed mostly in blood. These studies identified significant associations between cSVD pathology and endothelial dysfunction (ICAM-1 (refs. ^21–24^), VCAM-1 (refs. ^24–27^), E-selectin^24,28^, P-selectin^24^ and PGF^29,30^), inflammation (IL-6 (refs. ^31,32^), IL-18 (ref. ^32^), CRP^32^, TNFRSF11B^33^ and TNFRSF14 (ref. ^33^)), dysregulation of multiple components of the complement and IGF–IGFBP signaling pathways^34^, clotting pathway (vWF^33,35,36^), ECM breakdown (MMP-2 (ref. ^37^), MMP-9 (ref. ^38^), TIMP-1 (refs. ^37,39^) and HSPG2 (ref. ^33^)) and alteration of neuronal structural integrity (NEFL)^40^, many of which were validated in our study in CSF. We identify previously uncharacterized candidate biomarkers and provide an enriched expression profile that deepens the understanding of the pathophysiological framework of cSVD. We have uncovered both unique and shared patterns across cSVD conditions, with for example MMP12 emerging as an early and robust indicator. Our findings also highlight a critical role for arterial SMCs and shed light on the dynamics between disrupted metabolic, vascular, immune and neuronal mechanisms in cSVD. Furthermore, several key proteins were validated in plasma, with a subset improving 5-year risk stratification for cerebrovascular events and infarcts above and beyond current clinical benchmarks. In summary, we have identified proteins and biological processes present in cSVD that hold promise as therapeutic targets and biomarkers for these diseases.

Several proteins linked to ECM organization and SMC function were increased across CVD manifestations. In cSVD, in addition to ECM-related proteins such as MMP12 and ELN (further detailed below), matricellular proteins like POSTN and COMP were also altered. COMP, an ECM glycoprotein essential for collagen assembly and ECM stability, and has been linked to atherosclerosis, plaque area and vulnerability, and may induce tissue fibrosis^41^, whereas POSTN, upregulated in vascular injury, promotes ECM remodeling and inflammation^42,43^. In addition, the upregulation of PDLIM7 and ADAM8 suggests shared mechanisms in cSVD, involving ECM remodeling, vascular repair and altered cell-matrix communication. These findings point to a common cluster of upregulated proteins driving vascular integrity and remodeling in cSVD pathophysiology.

Among these shared proteins, MMP12 seems to be a key early player in the onset of cSVD, marked by an early and substantial increase, and association with WML progression. While previous research has shown associations between MMP2, MMP3 and MMP9 and vascular cognitive impairment^44,45^, we did not find an association with MMP3, and technical constraints prevented the evaluation of MMP2 (not present in the Olink panel) and MMP9 (above lower limit of detection (LOD) in <70% of individuals). Of note, we found an association of other MMPs with WMLs, such as MMP1, MMP7, MMP8 and MMP10. MMPs can influence cSVD by proteolyzing cerebrovascular basement membranes, altering tight junctions and activating bioactive molecules like clotting factors and signaling proteins^46^. MMP12 plays a key role in breaking down elastin and other ECM components^47^, which in pathological conditions may lead to compromised vessel wall integrity and elasticity. Increased MMP12 within the small arteries of individuals with cSVD further suggests its involvement in vessel wall remodeling and arterial stiffening, as supported by evidence from human aortic and animal studies^48^. This aligns with our observations of increased ELN levels across cSVD pathologies, suggesting the breakdown of elastic fibers and increased levels of various collagen types, especially with WMLs. Arterial stiffness can increase endothelial permeability, promote macrophage adhesion, and can lead to smooth muscle proliferation and vessel remodeling^49^. Additionally, MMP12 also modulates neuroinflammation through its effects on macrophages and neutrophils^50,51^, suggesting that its upregulation is linked to early vascular injury responses and subsequent pathogenic cascades. Notably, MMP12 has been identified as a genetic risk factor for large artery atherosclerosis^52^, supporting its broader role in vascular remodeling. Our findings now extend this role to SVD, positioning MMP12 as a potential shared effector across vascular territories.

MMP12 was co-abundant within a network of proteins that were increased in cSVD (module 5), whose levels increased during the early stages of WML and microbleed accumulation. This highlights the critical role of endothelial and SMC dysfunction at the onset of cSVD pathogenesis, with proteins likely involved in vascular remodeling. Such remodeling might include a shift toward a synthetic phenotype with reduced contractility and increased proliferation, as observed in animal models of hypertension^53^, and/or gradual degeneration and loss of SMCs^54^. The presence of cSVD-associated proteins like HSPB1, HSPB6, MMP12, PDLIM7, SERPINE1 and CSTB within this module suggests that oxidative stress, endothelial dysfunction, and vascular remodeling may collectively drive cSVD development and progression. Additionally, a strong association with module 10, which includes proteins like SMOC2 (refs. ^55,56^) (involved in ECM degradation and SMC migration) and VWF^57,58^ (a marker of endothelial dysfunction), points to a combined influence of metabolic and vascular dysfunction in promoting vascular damage and facilitating the entry of potentially harmful toxins and immune cells into the brain^59^. Recent genome-wide association studies (GWAS) have highlighted key biological pathways implicated in SVD, particularly those involving ECM remodeling and endothelial function^60^. Notably, our proteomic results converge on similar mechanisms, in particular reinforcing the emerging concept of cerebrovascular matrisome as central to SVD pathophysiology. The convergence of these pathways across genomic and proteomic levels supports a multifaceted model of cSVD involving both upstream genetic risk and downstream biological responses. Future integrative efforts combining proteomic and genetic data across all cSVD imaging markers may help further clarify upstream–downstream relationships and identify causal pathways that could be targeted for intervention. Furthermore, many circulating proteins implicated in cSVD likely reflect systemic vascular injury processes—such as endothelial dysfunction, inflammation, and oxidative stress—that are not exclusive to the brain. Vascular aging and atherosclerosis in peripheral organs, for example, have been linked to blood–brain barrier disruption and microvascular damage in the brain, supporting shared mechanisms across vascular beds^61^. While this broader vascular relevance may enhance our understanding of systemic contributors to cSVD, it may also limit brain-specific interpretation, particularly in plasma-based analyses; however, the detection and association of proteins with cSVD imaging markers in CSF adds evidence for a role in brain-specific pathophysiology. Future work comparing plasma and CSF signatures may help clarify the relative contributions of systemic and cerebral vascular processes. Finally, several proteins identified in our study, such as ELN, LTBP2, GDF15 and NEFL, have also been highlighted in recent proteomic aging signatures^62^. Their overlap suggests that molecular changes linked to cSVD may partially reflect shared mechanisms with biological aging, including vascular remodeling, inflammation and neuronal vulnerability.

The link between inflammation and vascular damage is well established, though the direction of causality remains debated^63^. WMLs were uniquely associated with VLMC- and perivascular fibroblast-enriched proteins, suggesting their role in immune cell recruitment, leukocyte migration and local inflammation through chemokine signaling and ECM production^64,65^. Our findings highlight immune cell involvement in WML progression, with strong associations observed for microglia- and perivascular macrophage-associated proteins, such as ITGAM, ITGB2, CHI3L1 and IL-15. This suggests a strong proinflammatory response, which might accelerate cell death, and the development and expansion of WMLs. Indeed, increased levels of serum CHI3L1 have recently been associated with cSVD and with the destruction of white matter macroscopic and microstructure, further associated with cognitive deficits^66^. In multiple sclerosis (MS), CHI3L1 was associated with neuronal deterioration, affirming its contribution to the progression of the disease^67^. In a rodent model, microglial inhibition via minocycline reduced white matter damage^68^, and its effects on microglia activation and BBB permeability in patients with cSVD are currently under investigation in a phase 2 clinical trial^69^. Future research should explore microglial states and stage-specific contributions to cSVD.

This study observed widespread downregulation related to neuron- and OPC-related proteins in WMLs, to a lesser extent in deep microbleeds and subcortical infarcts/lacunes, and notably lower levels in lobar microbleeds and cortical/cerebellar infarcts. This suggests that proteomic response to cerebrovascular injury may be highly localized, with subcortical regions and white matter more susceptible to disruptions and leading to a broader proteomic downregulation. Furthermore, WML-associated protein downregulation seems to have different impacts on cognitive outcomes. Specifically, a subset of proteins abundant in OPCs that were not linked to cognitive decline may be involved in the myelination process and serve broader roles, including proliferation, migration and differentiation of OPCs (ERBB3/ERBB4)^70^ and ECM organization (TIMP4)^71^. While these proteins are vital for maintaining efficient neuronal signaling, their direct impact on cognitive functions may not be immediate, and the brain may partially compensate for reductions in myelination efficiency.

Conversely, downregulated neuronal proteins, and some OPC-enriched proteins, are more directly linked to cognitive decline due to their role in synaptic function and plasticity. Key mediators include CBLN4 (synaptic organization and plasticity^72^), and NPTX1/NPTX2 (synaptic activity biomarkers and potential CSF biomarkers for synaptic dysfunction in neurodegeneration^73^). Additionally, EphA10 and EphB6, both pseudokinases of the Eph receptor family, are emerging but underexplored mediators of cognitive decline. While the Eph family is recognized for its involvement in synaptogenesis, synaptic plasticity and contribution to neurological diseases characterized by memory impairments^74^, EphA10 and EphB6 are not well characterized. Recent studies suggest EphB6 is present in both excitatory and inhibitory synapses^75^, highlighting its potential role in synaptic communication and the need for further investigation.

Several proteins upregulated in WMLs and mediating WML-associated cognitive decline also show increased levels in an AD context^76,77^, but remain associated with WML when adjusting for AD. Notable mediators include CSTB, a lysosomal cysteine protease implicated in neurodegeneration^78^ and NEFL, a key cytoskeletal component reflecting neuronal damage and associated with neurodegenerative diseases^79^. Other proteins such as SMOC2, MMP10 and TMSB10, associated with cognitive decline, highlight the role of ECM modulation and cell adhesion^55,80,81^ in cognitive function.

Given that the distinct topography of microbleeds is considered to reflect their underlying etiology, we revealed a compelling separation of molecular pathways. Deep microbleeds, commonly associated with hypertensive arteriosclerosis^82^, share a proteomic profile with other cSVD manifestations, including WMLs and subcortical infarcts/lacunes, with key proteins like MMP12, TNFRSF11B and SERPINE1 implicated in arterial stiffness and atherosclerosis pathogenesis^83–86^. By contrast, lobar microbleeds, linked to CAA, exhibit a distinct profile, with protein like ITGB2 and GLOD4 associated with amyloid pathology^87^. Nevertheless, proteins shared between lobar microbleeds and WMLs, such as CHIT1, CCN1 and MMP10, suggest overlapping pathways of vascular injury and tissue repair. These findings support a model in which deep microbleeds align with cSVD pathways, whereas lobar microbleeds parallel more closely amyloid pathways.

In the context of microbleeds, CHIT1 has emerged as a key protein, showing early and progressive increases with increasing microbleed burden. As a promising novel marker of acute or chronic inflammation, its expression has been mostly ascribed to microglia and infiltrating macrophages^88^. CHIT1 is increased in CSF in both AD^89,90^ and cerebrovascular dementia^91^, but its association with microbleeds persists after adjusting for AD pathology. Notably, in MS, baseline CSF CHIT1 levels reflect microglial activation early in the disease and correlated with subsequent disease progression^92^. Its early increase in microbleeds suggests that CHIT1 may serve as an early marker in the inflammatory response in microbleeds, warranting further investigation into its role in immune response and cSVD.

In cortical and cerebellar infarcts, which are indicative of large artery disease, RhoC emerged as a prominent marker. As a member of the Rho GTPase subfamily, RhoC regulates vascular homeostasis by modulating endothelial cell migration, proliferation, and permeability^93,94^, but remains unexplored in this context compared to RhoA and RhoB, which are upregulated in focal cerebral infarctions from as early as 2 days to as long as 38 months post-infarction^95^. In infarcts, particularly subcortical infarcts and lacunes, downregulation of astrocyte-enriched proteins, including RGMA, LRP1 and MEGF10, suggests altered astrocyte functions. These proteins are linked to reactive astrogliosis, scar formation after stroke^96^, and phagocytosis^97,98^, with LRP1 loss shown to mitigate demyelination and reverse WMLs.

After establishing key protein associations in CSF, we next focused on plasma proteomics to investigate whether similar patterns can be observed. We validated several proteins in plasma, with MMP7 emerging as top hit, showing strong associations with WMLs. Like MMP12, MMP7 plays a pivotal role in ECM degradation but is also critical for maintaining BBB integrity^99^. Increased MMP7 levels have been observed in conditions with BBB compromise, such as MS and traumatic brain injury, where its serum levels correlate with MRI-detected BBB dysfunction^100,101^. Given its role in ECM remodeling and BBB integrity, MMP7 has been proposed as a potential marker for BBB integrity, further underscoring its relevance in CVD.

To focus on the most robust and clinically relevant associations, we evaluated the predictive value of plasma proteins validated first in CSF, for cerebrovascular outcomes in the UK Biobank. This cross-fluid approach revealed a subset of plasma proteins that were associated with the risk of cerebrovascular events and significantly improved 5-year risk stratification, surpassing models based on age, sex and stroke risk score. Among the most prominent proteins were GDF15, WFDC2, NEFL and MMPs outlined previously. These findings align with a recent study demonstrating the utility of a protein-based risk score for 10-year infarct prediction^102^ but go further by linking specific proteins to cSVD-related changes and associating them with incident cerebrovascular outcomes. In line with earlier reports, higher circulating GDF15 has been associated with ischemic stroke severity, subclinical brain injury, white matter hyperintensity burden and future stroke^103–105^. Although GDF15 is a pleiotropic stress marker, its consistently strong associations with SVD-related imaging features and outcomes in our cohort reinforce its relevance for CVD and highlight its potential value in risk prediction. Together, these findings emphasize the predictive value of proteomic markers and underscores their potential to improve early risk identification and support personalized prevention strategies for cerebrovascular events and infarcts. Further research is needed to validate these findings across diverse populations and to explore the integration of proteomic biomarkers into clinical practice.

The cohort’s recruitment from southern Sweden, primarily composed of individuals of European descent narrows ethnic and racial representation. This points to the need for inclusion of more diverse populations in future studies, especially considering recent evidence of ancestry-specific protein co-abundance modules^106^ and findings that cSVD affects certain populations disproportionately^107^. While we define proteomic signatures across the different MRI markers, we acknowledge that many of the results are related to WMLs. Future studies with greater and more balanced representation of microbleed and infarct burden will be essential to further disentangle distinct signatures, characterize their longitudinal trajectories and better assess their clinical significance. Furthermore, to gain further insight into the relationship between CSF markers and cSVD progression, it is imperative to pursue longitudinal studies that track protein level changes over time.

The robustness and translational potential of our findings are supported by replication across multiple independent cohorts—BioFINDER-1, UK Biobank and ADNI—despite differences in demographics and proteomic platforms, including aptamer-based and reduced-panel assays. The consistency of associations across these diverse settings strengthens the generalizability and biological relevance of our results. Furthermore, many of the identified proteins exhibit druggable properties, suggesting they may be amenable to pharmacological targeting. Beyond DrugBank-listed compounds, several top targets are also being investigated in other ongoing drug development efforts. For example, MMP12 is targeted by selective investigational inhibitors such as Linvemastat (FP-020) and Aderamastat (FP-025), currently in development for inflammatory and fibrotic conditions^108^. Similarly, CHIT1 is the target of OATD-01, a chitinase inhibitor now in phase-2 trials for pulmonary sarcoidosis^109^. These examples illustrate opportunities to directly modulate biological pathways implicated in cSVD—an important step not only for exploring causal mechanisms but also for guiding the development of disease-modifying therapies.

In conclusion, using a multi-omics approach, we showed that cSVD manifestations present both shared and distinct proteomic signatures, revealing diverse pathophysiological profiles. These findings compel a re-evaluation of the traditional view of ‘cerebral small-vessel disease’ as an umbrella term toward a more nuanced classification into distinct ‘cerebral small-vessel diseases’, each characterized by unique biomarker profiles. We have highlighted a critical role for arterial SMCs in cSVD pathophysiology and identified candidate biomarkers in CSF and plasma, which hold promise for diagnostic and risk stratification applications. Furthermore, the identification of key molecular pathways and early biomarkers highlights opportunities for targeted therapeutic development, supporting efforts to improve the clinical management of cSVD.

## Methods

### Participants: BioFINDER-2 cohort

We included participants from the ongoing, prospective Swedish BioFINDER-2 cohort (NCT03174938). All provided written informed consent and were compensated per visit. Ethical approval was obtained from the Regional Ethical Committee in Lund, Sweden. All participants were recruited from south of Sweden at Skåne University Hospital or the Hospital of Ängelholm, Sweden, with data acquired between May 2017 and December 2023. Inclusion and exclusion criteria have been described previously^110^. The final dataset comprised 1,670 participants with complete baseline CSF proteomic and MRI data. Participants included cognitively unimpaired (intact cognition or subjective cognitive decline), mild cognitive impairment (MCI) and dementia due to AD or other neurodegenerative causes (for example PD and vascular dementia). The underlying etiology was established based on primary criteria for each condition^110^, either at baseline or during follow-up. Diagnoses followed DSM-5 criteria, with clinical AD defined as having a typical clinical syndrome of AD at the MCI or dementia stage, and AD biomarker confirmation, with either a positive result for CSF Aβ42/40 or Aβ-PET examination (visual read).

### MRI as measure of cerebrovascular pathology

MRI was performed using a 3 Tesla Siemens MAGNETOM Prisma scanner, equipped with a 64-channel receiver coil array (Siemens Healthcare). Imaging sequences included a whole-brain T1-weighted anatomical magnetization-prepared rapid gradient echo sequence (MPRAGE; TR = 1,900 ms, TE = 2.54 ms, voxel size of 1 × 1 × 1 mm^3^), a T2-weighted fluid-attenuated inversion recovery sequence (FLAIR; TR = 5,000 ms, TE = 393 ms, matching resolution with the T1-weighted image) and a 3D multi-gradient echo-pulse sequence (TR = 24 ms; TE = 5.00, 8.80, 12.60, 16.40 and 20.20 ms with monopolar/fly-back readout gradients, flip angle of 15°; voxel size of 0.8 × 0.8 × 1.4 mm^3^).

Following established criteria, microbleeds and infarcts were visually identified by an experienced neuroradiologist (D.W.), from T2* and FLAIR images, respectively^5,111^. Lesions were coded as 0 (absent) and ≥1 (present); for certain post hoc analysis, microbleed counts were log-transformed. Microbleeds were classified into lobar and deep, and infarcts into cortical/cerebellar or subcortical infarcts/lacunes. Classification of lesions was performed in accordance with the STRIVE-2 criteria^112^ (Standards for Reporting Vascular Changes on Neuroimaging), which define cSVD imaging markers. Among 138 individuals with subcortical infarcts/lacunes, 133 had lacunes and 5 had isolated subcortical infarcts; presumed of small vessel origin. WML were segmented using the SAMSEG tool (FreeSurfer v.7.1) on T1 and FLAIR images^113,114^. When treated as a continuous variable, WML volume was min–max scaled. In the main analysis, WML was treated as a binary variable to maintain uniformity in comparison to other cSVD. WML data were divided into tertiles, with the top tertile (0.497% of the intracranial volume (ICV)) representing individuals with increased WML burden (Supplementary Fig. 1), consistent with previous 0.5% ICV cutoff^115,116^. Baseline microbleed data were missing for 51 participants. MRI follow-ups were conducted 1, 2, 4 and 6 years after baseline measurement (mean ± s.d., 2.94 ± 1.02). The term cSVD refers to findings related to WMLs, microbleeds and subcortical infarcts/lacunes, whereas CVD denotes findings including cortical or cerebellar infarcts, or broader vascular pathologies.

### Description of comorbidities

As defined previously^117^, cardiovascular disease was defined as the presence of diagnosed hypertension, ischemic heart disease, or current use of antihypertensive or cardioprotective medication. Diabetes was defined as a diagnosis of either type 1 or type 2 diabetes. Hyperlipidemia was defined as diagnosed hyperlipidemia or current treatment with lipid-lowering medications.

### CSF proteomic measures

Baseline CSF samples were analyzed using Olink Explore 3072 (Proximity Extension Assay, PEA Technology)^118^, allowing for quantification of 2,943 proteins within eight multiplex protein panels, each containing 367–369 proteins (Oncology I/II, Neurology I/II, Cardiometabolic I/II and Inflammation I/II). Protein expression levels were reported as normalized protein expression values on a log_2_ scale, providing a relative quantification unit. While not reflecting absolute concentrations, this discovery-oriented approach is widely used for identifying group-level differences in large-scale proteomic studies. Samples failing quality control (QC) criteria were labeled with a warning. A LOD was defined as 3 × s.d. above the control-based background. Analysis focused on 1,388 proteins detected above LOD in ≥70% of participants^87^. Protein values with assay/QC warnings and outliers (±5 × s.d.) were excluded. For analysis, we retained all data points below LOD, following Olink recommendations^16,119^. To account for inter-individual variability in average CSF protein levels^120^, the average overall protein level (calculated as mean *z*-scored normalized protein expression values in highly detected proteins, *n* = 1,199, >90% above LOD) was included as a covariate. CSF p-tau217_Lilly_ concentrations were assessed using the Meso Scale Discovery immunoassay developed by Lilly Research Laboratories.

### Cell-type and functional enrichment analysis

Cell-type enrichment was assessed using two types of single-nucleus RNA-sequencing data: (1) the SEA-AD human MTG 10x atlas (166,868 nuclei from five donors; https://portal.brain-map.org/atlases-and-data/rnaseq/human-mtg-10x_sea-ad); and (2) the Human Brain Vascular Atlas^121^ (143,793 nuclei from the hippocampus and cortex from eight donors). Seurat objects were processed in Seurat (v.4.3.0) using AverageExpression to determine mean expression per cell type, after excluding ‘None’ annotations. We averaged expression across non-neuronal (microglia, astrocytes, oligodendrocytes, OPCs, vascular leptomeningeal cells and endothelial cells) and neuronal cells (GABAergic and glutamatergic neurons) in SEA-AD, and across major vascular and perivascular cell types in the vascular atlas (endothelial cells, SMCs (also distinguishing arterial and arteriolar SMCs), pericytes, astrocytes, macrophages, T cells and perivascular and meningeal fibroblasts). Percentage expression per cell type was then calculated. Statistical enrichment was evaluated using the EWCE^122^ package in R (v.1.6.0), with 10,000 bootstrap iterations against the 1,388 Olink proteins as background. The EWCE test assesses whether genes are more highly expressed in a given cell type than expected by chance, corresponding to a one-sided enrichment test.

Functional enrichment analysis for DAPs and modules was performed using WebGestalt^123^, with 1,388 Olink proteins as background set. We performed over-representation analysis with GO BP, CC and MF. Terms with *P*_FDR_ < 0.05 (Benjamini–Hochberg) were retained after redundancy reduction. For WML progression-associated DAPs, no significant terms remained after filtering, so results are shown without redundancy reduction.

### Co-abundance modules analysis

To identify modules of coexpressed proteins, we used the SpeakEasy2 consensus clustering algorithm, which simultaneously applies both top-down and bottom-up approaches to detect robust protein communities^20^. We utilized previously developed SpeakEasy clusters derived from 1,331 proteins^124^ and integrated additional proteins by correlating their expression values with cluster centroids, assigning them to the closest matching cluster on a winner-takes-all basis.

### Immunofluorescent staining from postmortem brain tissue

Frontal lobe white matter tissue was obtained from six individuals with SVD (age 81–90+ years; three women, severe white matter rarefaction (for example grade 3), ≥1 microscopic cortical infarct and Braak stage II–IV) and six non-SVD controls (age 85–90+ years; three women, low grade white matter rarefaction (for example grade 0–1), no infarcts and Braak stage II–IV) from the Arizona Study of Aging and Neurodegenerative Disorders and Brain and Body Donation Program at Banner Sun Health Research Institute^125^. The cases were evaluated according to the Cerebral White Matter Rarefaction grading system^126^ and National Institute on Aging and Reagan Institute (NIA-RI) criteria^127^, based on neurofibrillary tangles (NFT) Braak stages (I–VI)^128^ and Consortium to Establish a Registry for Alzheimer disease (CERAD) neuritic Aβ-plaque scores^129^.

Informed consent for the use of brain tissue, plasma and clinical data for research purposes was obtained from all participants or legal representatives in accordance with the International Declaration of Helsinki^130^. Procedures for the collection of brain tissue have been described previously^110,131^. The tissue collection protocols were approved by the Western Institutional Review Board of Puyallup, Washington, and the study was approved by the Swedish Ethical Review Authority. Directly after autopsy, brain tissue was fixed in 10% neutral buffered formalin solution containing 4% formaldehyde, followed by 48 h in 2% dimethyl sulfoxide/10% glycerol and another 48 h in 2% dimethyl sulfoxide/20% glycerol. The tissue was sectioned in 40-μm free-floating sections and stored in cryoprotectant at 4 °C until used for immunostaining.

To assess MMP12 localization by immunofluorescence, sections were stained with antibodies directed against MMP12 and laminin. Sections were incubated in blocking solution (5% goat serum and 0.25% Triton X-100 in KPBS) for 1 h, followed by mouse anti-laminin (1:200 dilution, clone 4C7, Dako, cat. no. C6198, lot 083M4778V) and rabbit anti-MMP12 (1:500 dilution, Biorbyt, cat. no. orb36364, lot E5525) in blocking solution for 48 h at 4 °C. After washing, sections were incubated with secondary antibodies (1:500 dilution, Invitrogen A11029, lot 2821059 goat anti-mouse and 1:500 dilution, Vector Laboratories DI-1549, lot ZH0421 goat anti-rabbit, Thermo Fisher Scientific) for 2 h at room temperature. All sections were treated with Sudan Black (1% in 70% ethanol, Sigma-Aldrich) for 5 min, rinsed, and mounted with Vectashield containing 4,6-diamidino-2-phenylindole (DAPI) (Vector Laboratories).

For quantification, five white matter arteries (outer diameter 150–400 μm) were imaged (×20, Olympus BX41). MMP12 area fraction within laminin-stained regions was quantified using Fiji/ImageJ software, as the thresholded MMP12-positive area divided by total laminin-positive area. The laminin-stained area was manually delineated as the region of interest and thresholds for each staining channel were adjusted manually based on the intensity distribution to ensure accurate separation of MMP12 and laminin signals. The observer conducting the thresholding and measurements was blinded. Group differences were analyzed using a Mann–Whitney *U*-test.

### Identification of drug targets

To assess the druggability of proteins identified in our analyses, we queried the DrugBank database^18^ (https://go.drugbank.com) using its advanced search function. As input, we used the gene names corresponding to the significant proteins from our study that were measured on the Olink platform. The output included descriptors such as Target Gene Name, Target UniProt, DrugBank ID, Drug Name, Biotech, Approved, Experimental, Investigational and Nutraceutical.

### Validation of key DAP in CSF in independent datasets

Two separate cohorts were used to validate key proteins identified in CSF in the BioFINDER-2 cohort: BioFINDER-1 and ADNI. The BioFINDER-1 study (registered under NCT01208675) consists of cognitively unimpaired and MCI individuals. The methodologies for participant selection and inclusion and exclusion criteria have been described previously^132^. In BioFINDER-1, proteomic analysis was conducted on a limited set of proteins from different panels, including Neuro-exploratory, Neurology, Inflammation and Cardiovascular-III, resulting in an overlap of 199 proteins with the main BioFINDER-2 cohort, used for further analysis. We selected participants who had proteomic and MRI data, specifically those for whom data on WMLs were available and segmented using SAMSEG (*n* = 383 individuals).

To validate key proteins externally, we used the ADNI dataset, an ongoing, multisite observation study (https://adni.loni.usc.edu/). We selected 729 participants from ADNI1, ADNI-GO and ADNI2 who had proteomic and MRI data, including WML information. Using SOMAScan 7K (v.4.1), an aptamer-based proteomics technology, protein levels of ~7,000 proteins were measured, and analyses were performed on 1,467 overlapping aptamers with BioFINDER-2. WML volume was taken from the ‘ST128SV’ variable, quantified using FreeSurfer from T1-weighted images. The total intracranial volume was taken from the variable ‘ST10CV’.

### Validation of key DAPs in plasma and predictive modeling in UK Biobank

We next validated key CSF proteins in plasma and assessed their relevance within a population-based setting. We first identified proteins that translate to plasma using BioFINDER-2 and further evaluated the predictive utility of these proteins for clinically relevant outcomes in the UK Biobank (application no. 105777)^133^. Plasma proteomics were conducted on BioFINDER-2 using SOMAscan 7k (*n*  = 1,599) and/or Olink Explore HT (*n*  = 694), with >95% participant overlap with the CSF dataset. QC and preprocessing matched CSF protocols, with additional log_2_ normalization applied to SOMAscan data. For each model (WML continuous and binary, deep and lobar microbleeds, subcortical infarcts/lacunes and cortical/cerebellar infarcts), we selected proteins significant in CSF and present in plasma. Associations were tested using linear models adjusted for age, sex and average protein levels, separately for each platform. Cross-platform concordance was assessed by Spearman correlation of 666 overlapping plasma proteins from 694 BioFINDER-2 participants. The correlation values and summary statistics for association with WMLs are provided in Supplementary Table 17. Given the larger dataset available for SOMAscan and modest concordance^134,135^, both datasets were used to maximize biomarker detection.

Proteins significantly associated with cSVD in plasma were further analyzed in UK Biobank, a large-scale, multicenter prospective cohort, where 2,922 plasma proteins were measured using Olink Explore 3072. Detailed information about the study’s methodology and data collection is available in the UK Biobank online protocol (www.ukbiobank.ac.uk). A total of 73 validated proteins in BioFINDER-2 were included for further analysis in UK Biobank, referred to as the ‘plasma cSVD set’. Associations with WMLs were examined in the UK Biobank, including 5,712 participants. WML volumes and ICV were obtained from the variables ‘Total volume of white matter hyperintensities from T1 and T2 FLAIR images’ and ‘Volume of Estimated Total IntraCranial (whole brain)’, respectively, with lesion segmentation performed using the BIANCA tool^136^.

We then evaluated the same proteins for association with incident outcomes: infarcts and cerebrovascular events, using a more comprehensive dataset, incorporating participants with proteomic data and diagnostic information. We split this dataset into a training (80%) and test (20%) subset to enable robust model development and validation. In the training set, Cox proportional hazard models were used to identify proteins associated with incident infarcts (ICD-10 I63) and cerebrovascular events (ICD-10 I60–I68, including hemorrhages, infarcts, arterial occlusions and stenosis, CAA and other CVDs), within 5 years, adjusting for age, sex and mean protein levels (R package survival v.3.4-0). These codes were obtained from a UK Biobank data field (41202) summarizing distinct primary or main diagnosis codes recorded in participants’ hospital inpatient records. Patients with a history of cerebrovascular events before their first visit were excluded from the analysis. The training set was used for feature selection, and the test set for evaluating machine-learning performance.

We next investigated whether these proteins improved prediction beyond established clinical risk factors based on the Stroke Risk Assessment Score^137^ from the American Stroke Association, including blood pressure, fasting glucose, BMI, total cholesterol, previous diagnoses of atrial fibrillation and diabetes mellitus, personal or family history of stroke, transient ischemic attack or heart attack, and smoking status. A composite risk score was calculated by assigning one point for each risk factor present: blood pressure >120/80 mm Hg, glucose >100 mg dl^−1^, BMI > 25 kg m^−^², cholesterol >160 mg dl^−1^, atrial fibrillation, diabetes, family or personal history of cerebrovascular events and smoking, with a maximum score of 8. Missing data were imputed (*k*-nearest neighbors for continuous variables and the median for categorical/binary variables).

Classification accuracy was tested using a random forest classifier. Hyperparameter tuning (max depth, 3, 5, 7, 10, 15, 20, 40, 50 and none) was performed on the training set with grid search and fivefold cross-validation, with balanced accuracy as the optimization metric. Performance in the test set was evaluated by the AUC. Proteins were modeled alone or alongside the stroke risk score or individual clinical variables to evaluate their added value in enhancing risk stratification and improving predictive accuracy. Statistical significance of the improvement in model performance was evaluated using DeLong’s test, comparing models to the base model containing age, sex and the stroke risk score. To assess feature contributions, Shapley additive explanations values were calculated for the model incorporating age, sex, clinical variables, proteins and average protein level.

### Statistics and reproducibility

Analyses were performed in R (v.4.2.1), Python (v.3.9.2) or GraphPad Prism (v.10.3.0). The main packages used were ggplot2 v.3.4.4, stats v.4.2.2, lme4 v.1.1-35.1, Seurat v.4.3.0 and EWCE v.1.6.0. No statistical methods were used to predetermine sample sizes but sample sizes were similar to or larger than previous publications^16,87^. The proteomic data were assumed to follow a normal distribution, although this was not formally tested. To ensure rigorous QC, samples from different diagnostic groups were approximately evenly distributed across plates, and each plate included relevant controls. All measurements were conducted by the company, with the operators blinded to any sample information. Linear models were used to assess DAP for each cSVD manifestation independently (increased WML burden versus low/absent WML burden, microbleeds+ versus microbleeds-, infarcts+ versus Infarcts-), adjusting for age, sex and average protein levels. Separate models distinguished lobar and deep microbleeds. Significant DAPs were re-tested in a combined model, including all cSVD markers, to determine whether the association persisted after adjusting for other cSVD. Only proteins retaining significance were considered in subsequent analyses. In selected analyses, models were additionally adjusted for clinical AD and PD diagnosis. In validation cohorts, WML volumes were analyzed as continuous variables, with covariates including age, sex, intracranial volume, mean overall protein levels and MRI-CSF interval (only in the UK Biobank, due to notable time gap).

Linear mixed-effect models, including random slope and intercept, were used to test the effects of CSF baseline protein levels on WML volume changes over time, using a protein × time interaction in the model. Age, sex, mean overall protein levels and intracranial volume (for WMLs only) were used as covariates. To investigate the relationship between protein modules and cSVD, we used linear models to analyze the association between average protein levels of each module and cSVD at baseline. For longitudinal assessments, linear mixed-effect models with a random slope and intercept, along with a module × time interaction were used to examine the effect of mean protein levels in each module at baseline on the changes of WML volume over time. Longitudinal analyses were not conducted for microbleeds and infarcts due to the limited number of cases showing progression over time, with even fewer cases for specific subtypes of microbleeds and infarcts. Trajectories of key proteins and modules with increasing WMLs and microbleed burden were visualized using a generalized additive model. All *P* values were corrected for multiple comparisons using the Benjamini–Hochberg FDR method (*P*_FDR_ < 0.05).

### Mediation analysis

We investigated whether WML-associated proteins mediated the relationship between WML volume (ICV-corrected) and executive function or mPACC rate of change using the mediation R package (v.4.5.0) with 1,000 bootstrapping iterations. All paths of the mediation model were controlled for age, sex, mean protein levels and education. The mPACC composite score was calculated using a mean of *z*-scores of the Alzheimer’s Disease Assessment Scale delayed recall (weighted double), animal fluency, Mini-Mental State Examination and SDMT. The executive function composite score was calculated as the mean of *z*-scores from the TMT-A, TMT-B and the SDMT, following methodology described previously^138,139^. To calculate the rate of change, linear mixed-effect models with a random slope and intercept were fitted, with executive function or mPACC as the dependent variable and time in years from the baseline scan as the independent variable. Association between baseline WML-associated proteins and cognitive decline (rate of change in executive function or mPACC) were assessed using linear models adjusted for the same covariates.

### Reporting summary

Further information on research design is available in the Nature Portfolio Reporting Summary linked to this article.

## Supplementary information

Supplementary Information Supplementary Figs. 1–11 and Supplementary Table Legends.

Reporting Summary

Supplementary Tables 1–17 Supplementary Tables 1–17.

Supplementary Data 1–11 Source data for Supplementary Figs. 1–11.

## Source data

Source Data Fig. 1 Statistical Source data.

Source Data Fig. 2 Statistical Source data.

Source Data Fig. 3 Statistical Source data.

Source Data Fig. 4 Statistical Source data.

Source Data Fig. 5 Statistical Source data.

Source Data Fig. 6 Statistical Source data.

Source Data Fig. 7 Statistical Source data.

Source Data Extended Data Fig. 2 Statistical Source data.

Source Data Extended Data Fig. 3 Statistical Source data.

Source Data Extended Data Fig. 4 Statistical Source data.

Source Data Extended Data Fig. 5 Statistical Source data.

Source Data Extended Data Fig. 6 Statistical Source data.

Source Data Extended Data Fig. 7 Statistical Source data.

Source Data Extended Data Fig. 8 Statistical Source data.

## Data Availability

Pseudonymized data from BioFINDER (Principal Investigator O.H.) will be shared upon reasonable request in a timely manner to qualified academic researchers and as long as data transfer is in agreement with EU legislation on the general data protection regulation and decisions by the Swedish Ethical Review Authority and Region Skåne, which will be regulated in a material transfer agreement. The agreement will specify how the data must be stored, protected and accessed, and define what the recipient can or cannot do. All planned analyses must comply with the approvals granted by the Swedish Ethical Review Authority. Source data with summary statistics have been uploaded on Zenodo at https://zenodo.org/records/17703751 (ref. ^140^). Data from the ADNI cohort used in this study can be accessed through the ADNI database (adni.loni.usc.edu), upon registration and agreement to the data use terms. Data from the UK Biobank cohort can be accessed upon application through the UK Biobank application process (ukbiobank.ac.uk), pending project approval and adherence to the data access terms. The snRNA-seq dataset from the Allen Brain Institute is openly accessible at https://portal.brain-map.org/atlases-and-data/rnaseq. The snRNA-seq dataset from the Human Brain Vascular Atlas can be found in the NCBI Gene Expression Omnibus (GEO) under accession code GSE163577.

## Code availability

All code was written using existing packages in R and Python and has been provided on GitHub at https://github.com/InesHrist/SVD_proteomics.
