# Supplementary material for: Identification of distinct and shared biomarker panels in different manifestations of cerebral small-vessel disease through proteomic profiling
Source: Nat Aging. 2026 Feb 24;6(3):703–21. doi: 10.1038/s43587-026-01081-7 (PMC13004690; doi:10.1038/s43587-026-01081-7)
Supplement: Supplementary file 1 — Supplementary Figs. 1–11 and Supplementary Table Legends. [file 43587_2026_1081_MOESM1_ESM.pdf]

nature aging

Supplementary information https://doi.org/10.1038/s43587-026-01081-7

Identification of distinct and shared biomarker panels in different manifestations of cerebral small-vessel disease through proteomic profiling

In the format provided by the authors and unedited

Supplementary Figures

Supplementary Fig. 1. Distribution of WML values and threshold for WML positivity

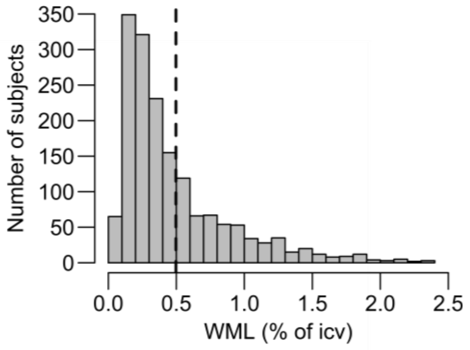

Legend: (a) Histogram depicting the distribution of WML values expressed as a percentage of intracranial volume (icv). A dashed line at 0.497 indicates the threshold for WML positivity. Participants in the lower two tertiles do not exhibit significant WML pathology, whereas those in the upper tertile are classified as exhibiting WML pathology.

Supplementary Fig. 2. Pathway analysis of common upregulated CVD-associated proteins

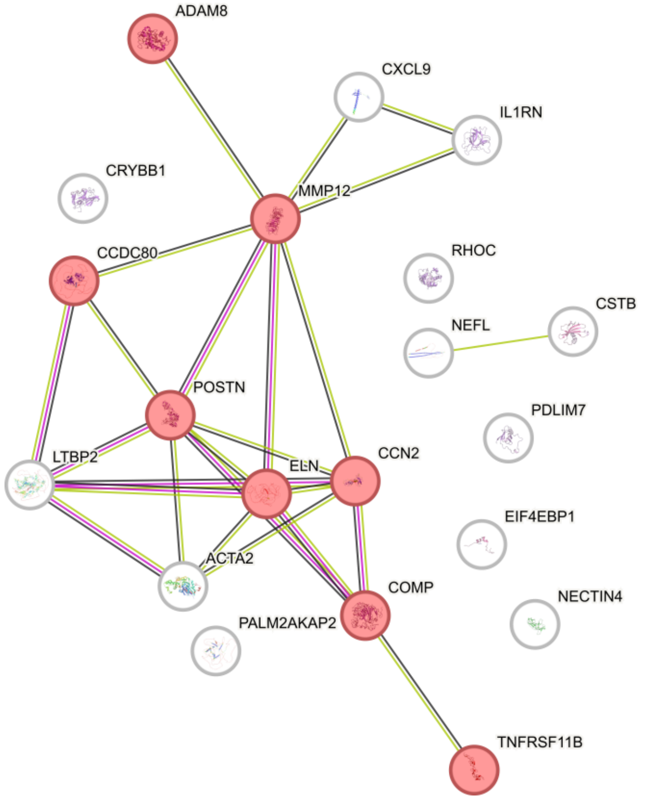

Legend: (a) Protein-protein interactions were identified using the STRING software with a minimum interaction score threshold of >0.4. Proteins highlighted in red are involved in extracellular matrix organization, a biological process that is significantly enriched (pFDR < 0.05) according to Gene Ontology (GO) analysis.

Supplementary Fig. 3. Differential protein expression when models for infarcts corrected for AD and PD diagnosis

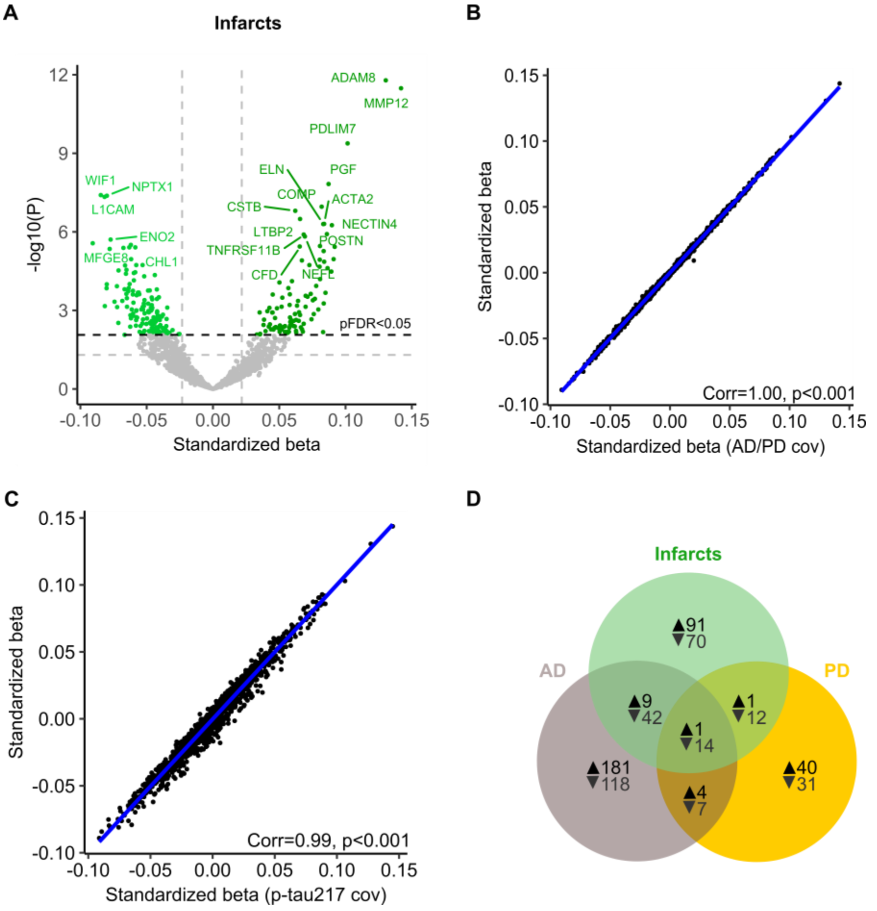

Legend: (a) Volcano plot showing differentially abundant proteins (DAPs) when comparing individuals with and without infarcts while correcting for AD and PD diagnosis. The models were adjusted for age, sex, and average protein level. The dashed lines represent significance threshold at α=0.05 before (gray) and after (black) FDR correction. Proteins below the p[FDR]<0.05 threshold were considered significant. For clarity, only the top 20 proteins are labeled. All standardized β coefficients are derived from two-sided linear regressions. (b) Comparison of the standardized betas from the main analysis assessing differential protein abundance vs. the standardized betas when further adding AD and PD diagnosis as covariates. (c) Comparison of standardized betas from the main analysis of differential protein abundance versus the betas obtained when additionally adjusting for continuous CSF p-tau217. For (b) and (c), the shaded band denotes the 95% confidence interval around the fitted linear regression line. For visual clarity, $p < 0.001$ is shown in the figure, although the exact two-sided p-values were $<2.2\times10^{-16}$. The p-values are reported in Supplementary Data Figure S3. (d) Venn diagram illustrating the overlap of differentially abundant proteins associated with infarcts and AD and PD, based on models mutually adjusted for the other conditions.

Supplementary Fig. 4. Sensitivity analyses across alternative lesion thresholds

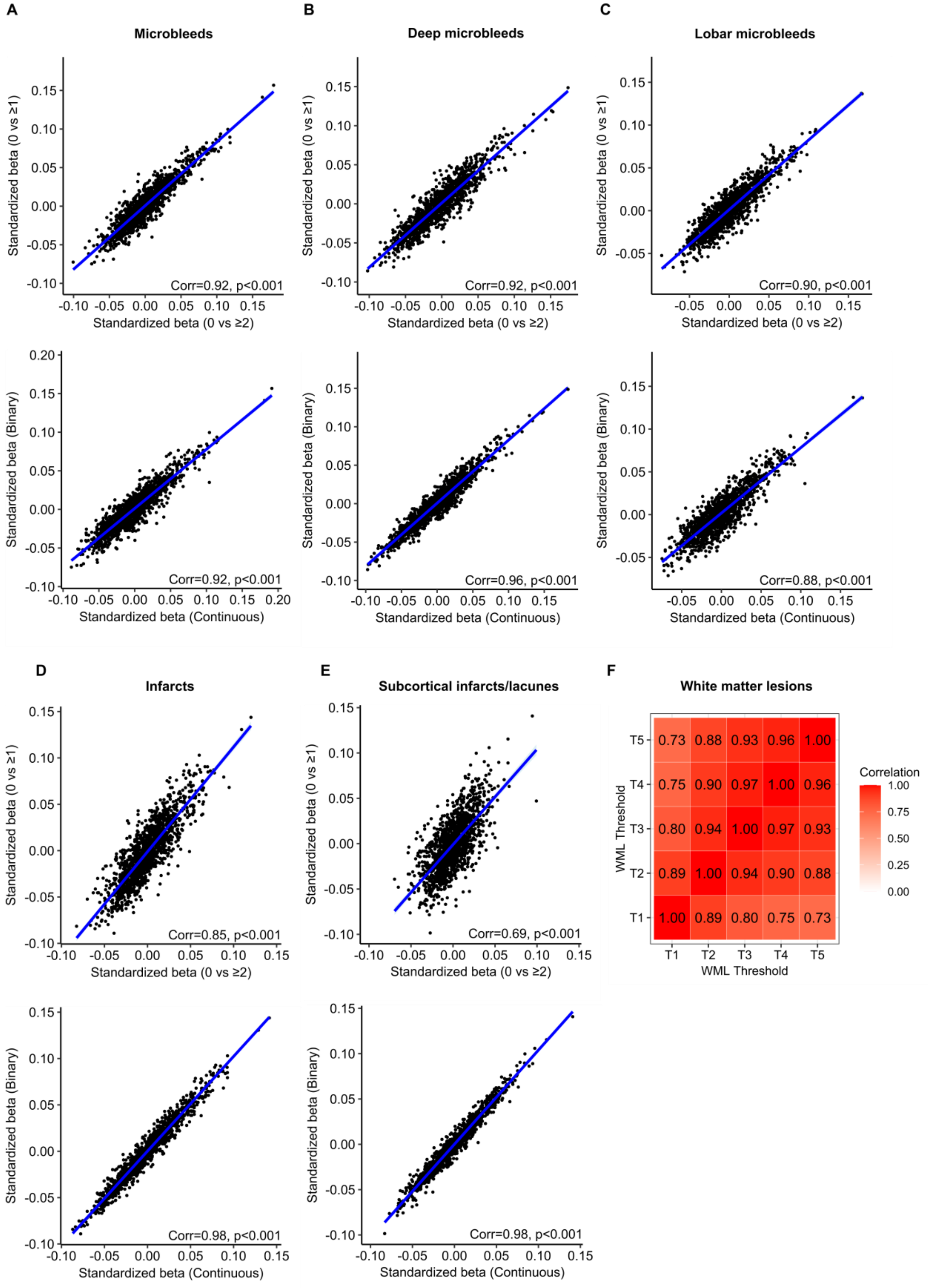

Legend: (a) Comparison of standardized betas from differential protein abundance analyses of all microbleeds using alternative definitions: binary classifications of 0 vs. ≥1 and 0 vs. ≥2

lesions (top panel), and binary (0 vs. ≥1, as used in the main analyses) versus continuous (log-transformed) lesion counts (bottom panel); (b) analysis restricted to deep or (c) lobar microbleeds. (d) Same analysis performed for infarcts, and restricted to (e) subcortical infarcts/lacunes. For all figures, the shaded band denotes the 95% confidence interval around the fitted linear regression line. For visual clarity, $p < 0.001$ is shown in the figure, although the exact two-sided p-values were $<2.2\times10^{-16}$. The p-values are reported in Supplementary Data Figure S4. (f) Heatmap showing pairwise correlations of standardized betas for differential protein analysis across five equally spaced binarization thresholds used to define elevated white matter lesion WML burden.

Supplementary Fig. 5. Impact of vascular risk factors and dementia adjustment on proteomic associations with SVD markers

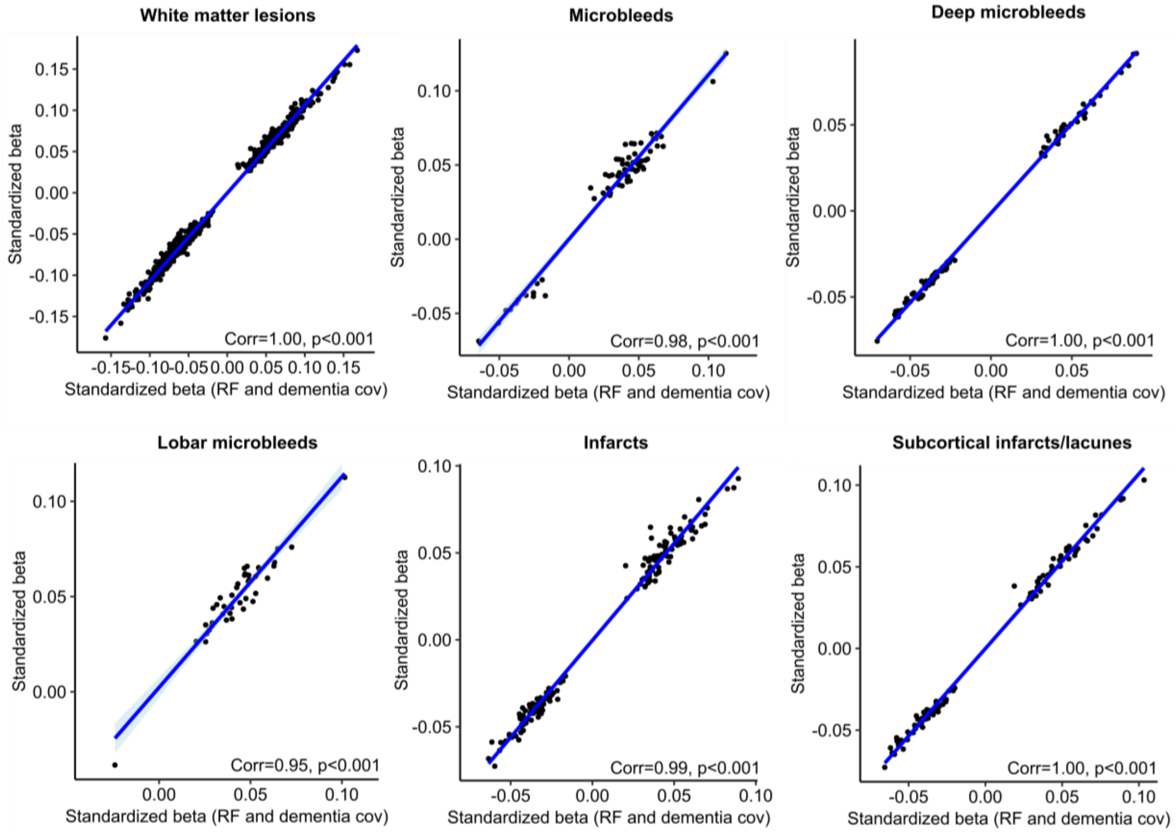

Legend: (a) Comparison of standardized beta coefficients from models with and without adjustment for vascular risk factors (cardiovascular disease, diabetes, hyperlipidemia) and dementia, for white matter lesions, microbleeds (deep and lobar), and infarcts, including separate analysis of subcortical infarcts/lacunes. For all figures, the shaded band denotes the 95% confidence interval around the fitted linear regression line. For visual clarity, $p < 0.001$ is shown in the figure, although the exact two-sided p-values were $<2.2\times10^{-16}$.

Supplementary Fig 6. Sensitivity analysis across cognitively unimpaired subjects

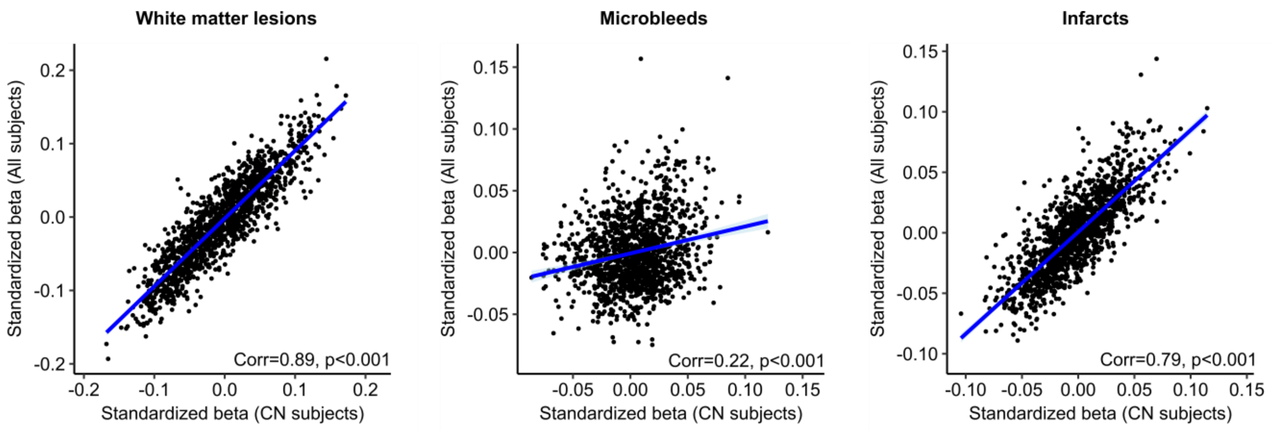

Legend: (a) Comparison of standardized betas from differential protein abundance analyses for white matter lesions, microbleeds and infarcts, restricted to cognitively unimpaired subjects. The shaded band denotes the 95% confidence interval around the fitted linear regression line. For visual clarity, $p < 0.001$ is shown in the figure, although the exact two-sided p-values were $<2.2\times10^{-16}$.

Supplementary Fig 7. Comparison of a modified SVD score in relation to lesion-specific proteomic signatures

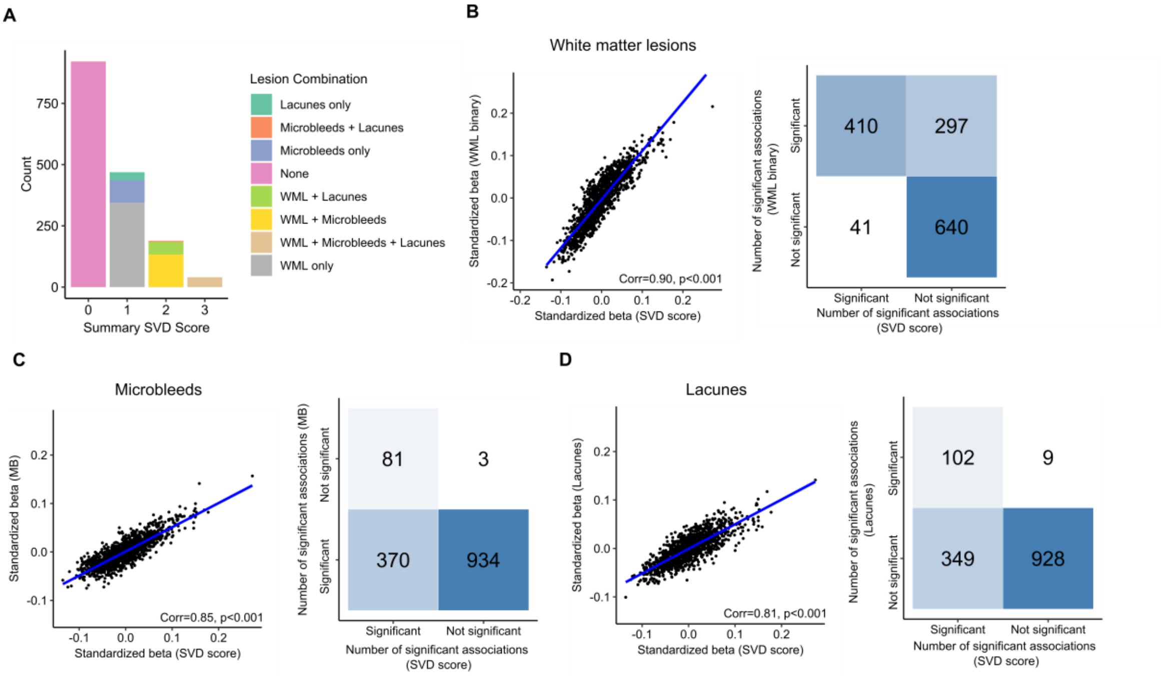

Legend: (a) Distribution of lesion combinations among participants based on a modified SVD score incorporating white matter lesions, microbleeds, and lacunes. (b) Comparison of standardized betas from the main analysis assessing differential protein abundance in relation to WML as a binary variable versus the SVD summary score, along with a confusion matrix showing the overlap in significance. (c) Comparison of standardized betas and significance overlap between MB-associated proteins and those identified using the SVD score. (d) Comparison of standardized betas and significance overlap between lacune-associated proteins and those identified using the SVD score, including a confusion matrix illustrating the agreement in significance status. For (b-d), the shaded band denotes the 95% confidence interval around the fitted linear regression line. For visual clarity, $p < 0.001$ is shown in the figure, although the exact two-sided p-values were $<2.2\times10^{-16}$.

Supplementary Fig. 8. Expression of upregulated DAP in arterial smooth muscle cells

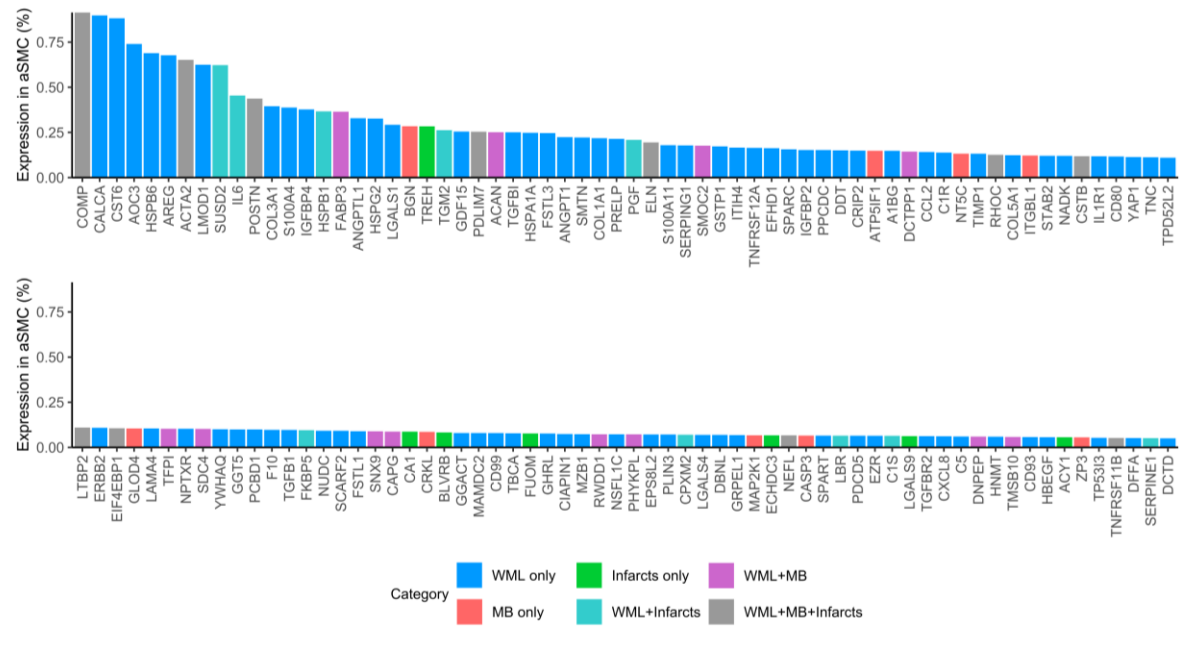

Legend: (a) Histograms showing the expression levels of upregulated DAP across all CVD in arterial smooth muscle cells, restricted to those with >5% expression in arterial smooth muscle cells based on the Human Brain Vascular Atlas.

Supplementary Fig. 9. Progression of WML pathology

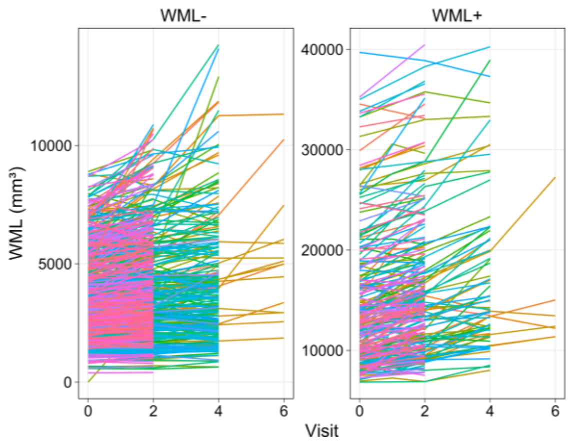

Legend: (a) Spaghetti plot illustrating the progression of WML volumes across successive visits. Individual trajectories represent WML volume change for each participant. For clarity, the plots are divided: the left panel (WML-) depicts subjects without WML pathology, while the right panel (WML+) shows subjects with WML pathology.

Supplementary Fig 10. Expression of DAP associated with WML progression in arterial smooth muscle cells

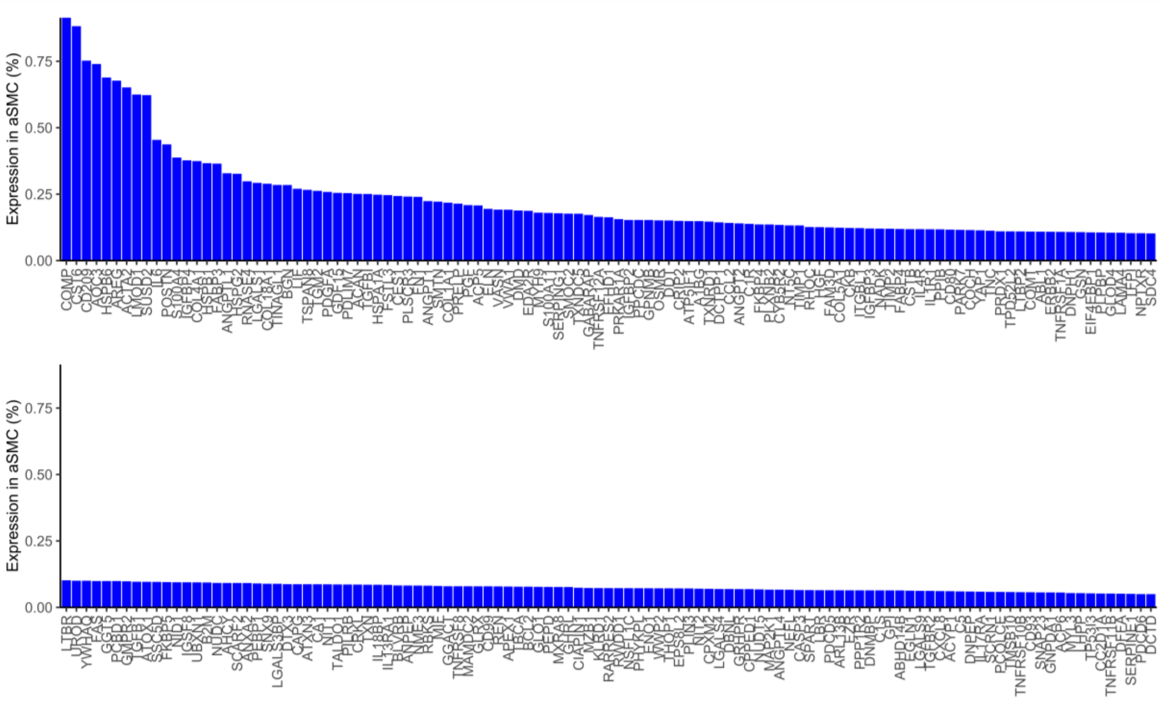

Legend: (a) Histograms showing the expression levels of DAP associated with WML progression in arterial smooth muscle cells, restricted to those with >5% expression in arterial smooth muscle cells based on the Human Brain Vascular Atlas.

Supplementary Fig 11. Cross-platform comparison of WML-associated proteins in BioFINDER-2 and ADNI

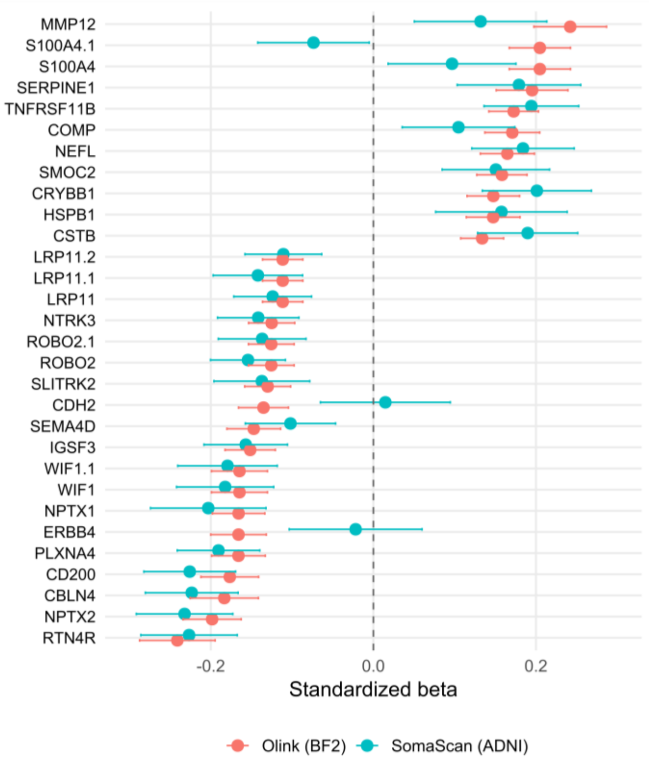

Legend: (a) Forest plots comparing standardized beta coefficients and 95% confidence intervals for the top 30 proteins (according to pFDR value in Olink in BioFINDER-2) associated with WML load, shown separately by assay platform: Olink in the BioFINDER-2 cohort and SomaScan in the ADNI cohort.

Supplementary Table Legends

Supplementary Table 1. BioFINDER-2 cohort demographics

Supplementary Table 2. Summary statistics of protein differential abundance across the different CVD categories

Supplementary Table 3. Drug targets for all DAP associated with white matter lesions, microbleeds and infarct subtypes

Supplementary Table 4. Gene Ontology significant terms from enrichment analysis in DAP in WML, MB and infarcts

Supplementary Table 5. Gene Ontology significant terms from enrichment analysis in the modules

Supplementary Table 6. Summary statistics of protein association with WML progression in the BioFINDER-2 cohort

Supplementary Table 7. Gene Ontology significant terms from enrichment analysis in DAP in longitudinal analysis

Supplementary Table 8. Summary statistics for mediation analysis of DAP on the association between WML and executive function rate of change

Supplementary Table 9. Demographics of validation cohorts: BioFINDER-1, ADNI and UK Biobank

Supplementary Table 10. Summary statistics of protein association with WML as a continuous variable in the BioFINDER-2 cohort

Supplementary Table 11. Summary statistics of DAP in the BioFINDER-1 cohort (validation cohort)

Supplementary Table 12. Summary statistics of DAP in the ADNI cohort (validation cohort)

Supplementary Table 13. Summary statistics of DAP in plasma in BioFINDER-2 using Olink Explore HT platform

Supplementary Table 14. Summary statistics of DAP in plasma in BioFINDER-2 using Somalogic 7k platform

Supplementary Table 15. Demographics of the train and test set in UK Biobank validation cohort

Supplementary Table 16. Summary statistics of Cox PH models in plasma in UK Biobank

Supplementary Table 17. Correlation and summary statistics for WML associations between SOMAscan and Olink protein measurements in plasma in the BioFINDER-2 cohort
