# Supplementary material for: Identification of distinct and shared biomarker panels in different manifestations of cerebral small-vessel disease through proteomic profiling
Source: Nat Aging. 2026 Feb 24;6(3):703–21. doi: 10.1038/s43587-026-01081-7 (PMC13004690; doi:10.1038/s43587-026-01081-7)
Supplement: Supplementary file 2 — Reporting Summary [file 43587_2026_1081_MOESM2_ESM.pdf]

nature portfolio

Corresponding author(s): Ines Hristovska & Oskar Hansson

Last updated by author(s): Oct 30, 2025

# Reporting Summary

Nature Portfolio wishes to improve the reproducibility of the work that we publish. This form provides structure for consistency and transparency in reporting. For further information on Nature Portfolio policies, see our Editorial Policies and the Editorial Policy Checklist.

## Statistics

For all statistical analyses, confirm that the following items are present in the figure legend, table legend, main text, or Methods section.

| n/a | Confirmed | |
|---|---|---|
| ☐ | ☒ | The exact sample size (*n*) for each experimental group/condition, given as a discrete number and unit of measurement |
| ☐ | ☒ | A statement on whether measurements were taken from distinct samples or whether the same sample was measured repeatedly |
| ☐ | ☒ | The statistical test(s) used AND whether they are one- or two-sided<br>*Only common tests should be described solely by name; describe more complex techniques in the Methods section.* |
| ☐ | ☒ | A description of all covariates tested |
| ☐ | ☒ | A description of any assumptions or corrections, such as tests of normality and adjustment for multiple comparisons |
| ☐ | ☒ | A full description of the statistical parameters including central tendency (e.g. means) or other basic estimates (e.g. regression coefficient) AND variation (e.g. standard deviation) or associated estimates of uncertainty (e.g. confidence intervals) |
| ☐ | ☒ | For null hypothesis testing, the test statistic (e.g. $F$, $t$, $r$) with confidence intervals, effect sizes, degrees of freedom and $P$ value noted<br>*Give P values as exact values whenever suitable.* |
| ☒ | ☐ | For Bayesian analysis, information on the choice of priors and Markov chain Monte Carlo settings |
| ☒ | ☐ | For hierarchical and complex designs, identification of the appropriate level for tests and full reporting of outcomes |
| ☐ | ☒ | Estimates of effect sizes (e.g. Cohen's $d$, Pearson's $r$), indicating how they were calculated |

*Our web collection on statistics for biologists contains articles on many of the points above.*

## Software and code

Policy information about availability of computer code

| | |
|---|---|
| Data collection | No software was used. |
| Data analysis | R version 4.2.1 and python version 3.9.2 were used for analyses. No custom algorithm or software was used.<br>Main packages used were ggplot2 v3.4.4, stats v.4.2.2, lme4 v1.1-35.1, Seurat v4.3.0, EWCE v1.6.0. |

For manuscripts utilizing custom algorithms or software that are central to the research but not yet described in published literature, software must be made available to editors and reviewers. We strongly encourage code deposition in a community repository (e.g. GitHub). See the Nature Portfolio guidelines for submitting code & software for further information.

## Data

Policy information about availability of data

All manuscripts must include a data availability statement. This statement should provide the following information, where applicable:

- Accession codes, unique identifiers, or web links for publicly available datasets
- A description of any restrictions on data availability
- For clinical datasets or third party data, please ensure that the statement adheres to our policy

BioFINDER data are available from the principal investigator (OH), pseudonymized data will be shared by request from a qualified academic investigator as long as data transfer is in agreement with EU legislation on the general data protection regulation and decisions by the Ethical Review Board of Sweden and Region Skåne, which should be regulated in a data transfer agreement.

## Research involving human participants, their data, or biological material

Policy information about studies with human participants or human data. See also policy information about sex, gender (identity/presentation), and sexual orientation and race, ethnicity and racism.

| | |
|---|---|
| Reporting on sex and gender | Sex was self-reported and was included as a covariate in all analyses. |
| Reporting on race, ethnicity, or other socially relevant groupings | Race and ethnicity were not included as confounding factors in this manuscript. The BioFINDER-2 cohort is relatively homogeneous with a vast majority of White individuals from south of Sweden. |
| Population characteristics | Detailed information is given in Supplementary Table 1. |
| Recruitment | In BioFINDER-2, the sample consisted of patients that had been referred to participting memory clinics (mostly from primary care) and most cognitively unimpaired participants were recruited from the general population in the south of Sweden. Informed consent was obtained from all participants and they were compensated for each study visit they completed. |
| Ethics oversight | Ethical approval was given by the Regional Ethical Committee of Lund University. |

Note that full information on the approval of the study protocol must also be provided in the manuscript.

# Field-specific reporting

Please select the one below that is the best fit for your research. If you are not sure, read the appropriate sections before making your selection.

☒ Life sciences ☐ Behavioural & social sciences ☐ Ecological, evolutionary & environmental sciences

For a reference copy of the document with all sections, see nature.com/documents/nr-reporting-summary-flat.pdf

# Life sciences study design

All studies must disclose on these points even when the disclosure is negative.

| | |
|---|---|
| Sample size | We did not a priori perform a sample size calculation for this study. We selected the largest sample size available with participants who had CSF proteomics as well as measures of cerebral small vessel disease pathology, for a total of 1670 participants. |
| Data exclusions | Proteins for which more than 70% of participants had measurements below the limit of detection were excluded and not considered for analyses. This exclusion criteria resulted in 1388 proteins analyzed. |
| Replication | We used three independent cohorts to validate the main proteomic hits in relation to white matter lesions (measures of microbleeds and infarcts were not available in these cohorts): BioFINDER-1 with Olink and ADNI with SomaLogic proteomics in CSF samples and UK Biobank with Olink proteomics in plasma samples. Main proteomic hits in relation to all cSVD were assessed for validation in plasma in the same cohort, BioFINDER-2, in two different platforms (Olink and Somalogic). |
| Randomization | We categorized subjects into different groups based on the presence or absence of various small vessel disease manifestations, including white matter lesions, microbleeds, and infarcts, and their subtypes. |
| Blinding | Proteomic measurements were performed blinded to any demographics or clinical characteristics. |

# Reporting for specific materials, systems and methods

We require information from authors about some types of materials, experimental systems and methods used in many studies. Here, indicate whether each material, system or method listed is relevant to your study. If you are not sure if a list item applies to your research, read the appropriate section before selecting a response.

### Materials & experimental systems

| n/a | Involved in the study | |
|---|---|---|
| ☐ | ☒ | Antibodies |
| ☒ | ☐ | Eukaryotic cell lines |
| ☒ | ☐ | Palaeontology and archaeology |
| ☒ | ☐ | Animals and other organisms |
| ☐ | ☒ | Clinical data |
| ☒ | ☐ | Dual use research of concern |
| ☒ | ☐ | Plants |

### Methods

| n/a | Involved in the study | |
|---|---|---|
| ☒ | ☐ | ChIP-seq |
| ☒ | ☐ | Flow cytometry |
| ☐ | ☒ | MRI-based neuroimaging |

# Antibodies

| | |
|---|---|
| Antibodies used | Details about antibody-based proteomic technology is provided by Olink, with all details found here: https://www.olink.com/content/uploads/2021/09/olink-white-paper-pea-a-high-multiplex-immunoassay-technology-with-qpcr-or-ngs-readout-v1.0.pdf<br>For immunostainings, we used MMP12 (1:500, Biorbyt, cat # orb36364, Lot E5525), Laminin (1:200, clone 4C7, Dako, cat # C6198, Lot 083M4778V), Goat anti-rabbit (1:500, Vector Laboratories DI-1549, lot ZH0421) and Goat anti-mouse (1:500, Invitrogen A11029, Lot 2821059). |
| Validation | Extensive validation has been conducted by Olink, with all details of the Explore 3072 available here: https://olink.com/content/uploads/2022/10/olink-explore-validation-data.pdf |

# Clinical data

Policy information about clinical studies
All manuscripts should comply with the ICMJE guidelines for publication of clinical research and a completed CONSORT checklist must be included with all submissions.

| | |
|---|---|
| Clinical trial registration | BioFINDER-2: NCT03174938, BioFINDER-1: NCT01208675 |
| Study protocol | Please see www.biofinder.se |
| Data collection | BioFINDER-2 participants include a mix of population-based and memory clinic-based studies in Lund and Malmö, in Sweden and all imaging data was acquired at Skane University Hospital between April 2017 and December 2023. |
| Outcomes | The predefined primary outcome measures were the differentially expressed proteins between the different cerebral small vessel disease manifestations. After having identified the differentially expressed proteins for each category, we evaluated different outcomes based on 1) functional enrichment analyses, 2) cell-type enrichment analyses, 3) co-abundance modules, 4) associations with WML progression, 5) mediation analysis on association between WML and cognitive decline, and 6) validation in CSF and extensive validation in plasma. |

# Plants

| | |
|---|---|
| Seed stocks | *Report on the source of all seed stocks or other plant material used. If applicable, state the seed stock centre and catalogue number. If plant specimens were collected from the field, describe the collection location, date and sampling procedures.* |
| Novel plant genotypes | *Describe the methods by which all novel plant genotypes were produced. This includes those generated by transgenic approaches, gene editing, chemical/radiation-based mutagenesis and hybridization. For transgenic lines, describe the transformation method, the number of independent lines analyzed and the generation upon which experiments were performed. For gene-edited lines, describe the editor used, the endogenous sequence targeted for editing, the targeting guide RNA sequence (if applicable) and how the editor was applied.* |
| Authentication | *Describe any authentication procedures for each seed stock used or novel genotype generated. Describe any experiments used to assess the effect of a mutation and, where applicable, how potential secondary effects (e.g. second site T-DNA insertions, mosiacism, off-target gene editing) were examined.* |

# Magnetic resonance imaging

## Experimental design

| | |
|---|---|
| Design type | *Indicate task or resting state; event-related or block design.* |
| Design specifications | *Specify the number of blocks, trials or experimental units per session and/or subject, and specify the length of each trial or block (if trials are blocked) and interval between trials.* |
| Behavioral performance measures | *State number and/or type of variables recorded (e.g. correct button press, response time) and what statistics were used to establish that the subjects were performing the task as expected (e.g. mean, range, and/or standard deviation across subjects).* |

## Acquisition

| | |
|---|---|
| Imaging type(s) | Structural MRI |
| Field strength | 3T |
| Sequence & imaging parameters | All details are in the Methods section, and in the references therein. |
| Area of acquisition | Whole brain |

Diffusion MRI ☐ Used ☒ Not used

## Preprocessing

| | |
|---|---|
| Preprocessing software | FreeSurfer version 7.1 |
| Normalization | Volume of white matter lesion (SAMSEG) normalized by intracranial volume |
| Normalization template | Data extracted from single-subject space. |
| Noise and artifact removal | n.a. |
| Volume censoring | n.a. |

## Statistical modeling & inference

| | |
|---|---|
| Model type and settings | All details are in the Methods section |
| Effect(s) tested | Presence and volume of white matter lesions, presence of microbleeds and infarcts were used as predictors. |

Specify type of analysis: ☒ Whole brain ☐ ROI-based ☐ Both

| | |
|---|---|
| Statistic type for inference (See Eklund et al. 2016) | n.a. |
| Correction | FDR correction |

## Models & analysis

| n/a | Involved in the study |
|---|---|
| ☒ | ☐ Functional and/or effective connectivity |
| ☒ | ☐ Graph analysis |
| ☐ | ☒ Multivariate modeling or predictive analysis |

| | |
|---|---|
| Multivariate modeling and predictive analysis | Use of MRI data to investigate relationship between cerebral small vessel disease and protein levels. White matter lesions, microbleeds and infarcts are used as independent variables, and proteins as outcomes, along with the covariates. |
